# Characterization of gene regulation and protein interaction networks for Matrin 3 encoding mutations linked to amyotrophic lateral sclerosis and myopathy

**DOI:** 10.1038/s41598-018-21371-4

**Published:** 2018-03-06

**Authors:** M. Carolina Gallego Iradi, Judy C. Triplett, James D. Thomas, Rachel Davila, Anthony M. Crown, Hilda Brown, Jada Lewis, Maurice S. Swanson, Guilian Xu, Edgardo Rodriguez-Lebron, David R. Borchelt

**Affiliations:** 1 Department of Neuroscience, Center for Translational Research in Neurodegenerative Disease, McKnight Brain Institute, University of Florida, Gainesville, FL USA; 2 Department of Molecular Genetics & Microbiology, Center for Neurogenetics, Genetics Institute, University of Florida, Gainesville, FL USA; 3 Department of Pharmacology & Therapeutics, Center for Translational Research in Neurodegenerative Disease, McKnight Brain Institute, University of Florida, Gainesville, FL USA

## Abstract

To understand how mutations in *Matrin 3* (*MATR3*) cause amyotrophic lateral sclerosis (ALS) and distal myopathy, we used transcriptome and interactome analysis, coupled with microscopy. Over-expression of wild-type (WT) or F115C mutant MATR3 had little impact on gene expression in neuroglia cells. Only 23 genes, expressed at levels of >100 transcripts showed ≥1.6-fold changes in expression by transfection with WT or mutant MATR3:YFP vectors. We identified ~123 proteins that bound MATR3, with proteins associated with stress granules and RNA processing/splicing being prominent. The interactome of myopathic S85C and ALS-variant F115C MATR3 were virtually identical to WT protein. Deletion of RNA recognition motif (RRM1) or Zn finger motifs (ZnF1 or ZnF2) diminished the binding of a subset of MATR3 interacting proteins. Remarkably, deletion of the RRM2 motif caused enhanced binding of >100 hundred proteins. In live cells, MATR3 lacking RRM2 (ΔRRM2) formed intranuclear spherical structures that fused over time into large structures. Our findings in the cell models used here suggest that MATR3 with disease-causing mutations is not dramatically different from WT protein in modulating gene regulation or in binding to normal interacting partners. The intra-nuclear localization and interaction network of MATR3 is strongly modulated by its RRM2 domain.

## Introduction

A dominantly inherited mutation in *MATR3* (S85C) was first described in a large, multigenerational family, with slowly progressing (15 years) asymmetrical myopathy and concomitant vocal cord paralysis^1^. A later study confirmed the S85C mutation as causing myopathy^2^. Two dominant mutations in *MATR3* (F115C and T622A), however, were discovered by exome sequencing in patients with classic ALS^3^. The rarity of MATR3 cases has limited investigations of human pathology. Johnson and colleagues described intensified nuclear staining of MATR3 in sporadic ALS cases, with some cells showing diffuse cytoplasmic accumulation^3^. In cultured cells, the majority of WT and mutant MATR3 is localized to the nucleus^4^. MATR3 was found to co-immunoprecipitate with TDP-43 in cultured cells, but there was minimal TDP-43 pathology in MATR3 patient tissues^3^. A recent study, however, has found evidence that a subset of TDP-43 immunoreactive inclusions in sporadic ALS cases are also immunoreactive for MATR3^5^. Disease-causing mutations in TDP-43 generally cause catastrophic changes in the biology of the protein such that it accumulates in the cytoplasm and forms inclusions [for review see^6^]. ALS mutations in FUS similarly cause catastrophic changes in localization and solubility, shifting from nuclear to cytoplasmic compartments to form inclusions^6^. For MATR3, the available data have not indicated that disease mutations produce similar severe alterations in localization^3,7^.

MATR3 was initially identified in rat liver as a component of what was termed the nuclear scaffold or matrix^8^. The 847 amino acid sequence of MATR3 contains multiple recognizable motifs including a nuclear localization signal (NLS), two C2H2-type zinc finger (ZnF) binding domains, and 2 RNA recognition motifs (RRM)^8^. The two RNA binding domains in MATR3 are homologous to those found in the heterogeneous nuclear ribonucleoproteins (HNRNPs) L and I, and both appear functional in electrophoretic mobility shift assays^9^. MATR3 is heavily modified by phosphorylation, sumoylation, acetylation, and ubiquitination {http://www.uniprot.org/uniprot/P43243} {www.phosphosite.org}. How these modifications modulate function has not been extensively studied. Multiple functions have been described for MATR3, including DNA binding^8^ and transcriptional regulation^10–12^. Although RRM domains may bind DNA or RNA, there is ample evidence that MATR3 may function primarily in RNA processing. MATR3 can interact in an RNA-dependent manner with the heterogeneous ribonuclear protein HNRNPK and the RNA helicase DHX9^13^. The same group identified several potential mRNA substrates for MATR3 binding, and suggested that binding may stabilize these mRNAs. Other studies have identified MATR3 in complexes involved in the retention of hyper-edited RNA in the nucleus^14^ and in miRNA-induced silencing of expression as part of the Argonaute complexes^15^. MATR3 modulates the propagation of human immunodeficiency virus (HIV), in conjunction with the HIV REV protein, by facilitating the cytoplasmic accumulation and translation of viral mRNA^16^. Additionally, MATR3 has been reported to regulate alternative splicing of hundreds of genes with a subset of these splicing events co-regulated by interaction with PTBP1 (polypyrimidine tract binding protein)^17^.

A previous study has examined the interactome of WT human MATR3 by expressing tagged MATR3 in human HeLa cells, identifying numerous interactors including multiple splicing factors (heterogeneous nuclear ribonucleoproteins) that play roles in mRNA processing, metabolism, and transport^18^. A more recent study of MATR3 interactomes included analysis of WT and mutant MATR3 in murine NSC-34 cells^19^. This later study, identified proteins of the TRanscription and EXport (TREX) complex as interacting partners that may be influenced by disease-causing mutations. It was not clear in either of these studies whether these interactions were RNA-dependent or RNA-independent. Other proteins identified in these studies include transcriptional regulators of ILF2/ILF3 and factors involved in RNA editing such as double-stranded specific adenosine deaminase (ADAR)^18^. MATR3 has also been identified in proteomic screens as interacting with TDP-43^20^, and other proteins that interact with TDP-43 including FUS, HNRNPA1 and HNRNPA2B1^18^. These four RNA binding proteins have all also been associated with ALS or multisystemic proteinopathy^6,21–23^. Finally, MATR3 has been identified as part of large complex of splicing regulators that includes Rbfox proteins^24^.

In the present study, we have used three complementary approaches to characterize the impact that disease-causing mutations in *MATR3* may have on its ability to regulate gene expression, interact with other proteins, or properly localize within the cell. In a transcriptomic study of H4 neuroglioma cells transiently expressing either WT or F115C MATR3 fused to yellow fluorescent protein (YFP), we found little difference between the RNA profiles of cells expressing WT and mutant MATR3. Compared to control cells expressing YFP alone, relatively few genes showed changes in gene expression of ≥1.6-fold and, there was only one RNA that we could identify as potentially showing a change in splicing when MATR3 was over-expressed. Our study of the interactome of WT and mutant MATR3 similarly yielded little evidence of obvious differences in protein:protein interactions. Our study did not identify proteins of the TREX complex as being prominent interactors with WT or mutant MATR3. Our interaction network study differs from the recently published effort^19^ in that here we use human cells and we over-express the MATR3 variants. We also use a different affinity capture paradigm in which the expressed MATR3 proteins encode a biotinylation tag sequence (AviTag)^25,26^. This approach produced highly reproducible data that point towards MATR3 as a modulator of RNA processing with the mutant protein being correctly integrated within this network. Collectively, our data suggest that disease-causing mutations in MATR3 do not produce dramatic abnormalities in the ability of MATR3 to regulate gene expression or interact with normal binding partners. Through analysis of MATR3 proteins with deletions in conserved RNA recognition motifs (RRMs) and Zn finger motifs (ZnFs), we revealed a crucial role of the RRM2 motif in MATR3 in regulating its interaction with other nuclear proteins and maintaining its intranuclear localization.

## Results

To examine the relative ability of WT and mutant (F115C) MATR3 to regulate gene expression and RNA processing, we transfected human H4 neuroglioma cells with WT human MATR3 fused to yellow fluorescent protein (WT-MATR3:YFP), F115C-MATR3:YFP, and YFP alone. Although not ideal, the neuroglioma cells provide a cell model to assess human MATR3 regulation of human gene expression. At 24 hours post-transfection, we used fluorescence activated cell sorting (FACS) to isolate the transfected cells from untransfected cells and ultimately isolated RNA from 4 cell populations; WT-MATR3:YFP, F115C-MATR3:YFP, and YFP expressing with a non-transfected cell control. The transfections were done in triplicate and with three independent isolates of cells from the FACS. The cells expressing each construct were isolated based on similar fluorescence intensity thresholds to normalize for protein expression, with each FACS run isolating the same total number of cells. After removal of ribosomal RNA, equal amounts of RNA isolated from these cells, from each batch of FACS isolation, were each subjected to high-density RNA-seq analysis, yielding triplicate sets of expression data for >23,000 genes from each population of cells (Supplemental Table S1). Using RPKM (Reads Per Kilobase of transcript per Million) values calculated for each of these ~23,000 genes, unsupervised clustering of Pearson correlation values for all pair-wise comparisons was performed (Supplemental Figure S1), and this analysis demonstrated high concordance in global gene expression between individual replicates for each treatment group. For example, the maximum RPKM numbers for MATR3 in each replicate data set of the four conditions were as follows; 1036, 1019, and 1085 RPKM in untransfected cells; 746, 666, and 748 RPKM in cells expressing YFP alone (statistically significant suppression; adj. p < 0.01); 20755, 27346, and 52559 RPKM in cells expressing WT-MATR3:YFP; and 35818, 27855, and 34816 in cells expressing F115C-MATR3:YFP (difference between WT and mutant not significant; adj. p < 0.63)(Supplemental Table S1). The estimated fold induction of WT and mutant MATR3:YFP mRNA over endogenous levels MATR3 mRNA was 30-fold on average.

Transfection of these cells produced a large number of changes in gene expression. For example, compared to untransfected cells, the cells expressing YFP alone showed ≥2-fold changes in the expression of ~3,000 genes (1073 genes upregulated and 1,957 genes down-regulated; adj. p < 0.01) (Supplemental Table S1). A similar number of changes occurred in cells expressing WT-MATR3:YFP (1520 genes upregulated and ~2122 genes down regulated by ≥2-fold) as compared to untransfected cells (Supplemental Table S1). However, most of these genes were expressed at low levels (<10 maximum per base reads).

To probe the data for insight into the function of WT- or F115C-MATR3 in regulating gene expression, we compared data from cells expressing YFP alone to cells expressing WT-MATR3:YFP. Relative to cells expressing YFP, there were 224 genes in cells expressing WT-MATR3:YFP that were upregulated and 242 genes that were downregulated by ≥2-fold (adj. p < 0.05) (Supplemental Table S1). When we filtered these data to focus on genes that were not induced or suppressed by expression of YFP alone, there were 23 genes that specifically showed changes in expression of ≥1.6-fold when WT-MATR3:YFP was expressed (Table 1). Five coding genes, and one non-coding miRNA, were identified as upregulated and 17 coding genes showed down-regulation (Table 1; Supplemental Table 1). None of these genes showed statistically significant differences in expression between cells expressing WT-MATR3:YFP and F115C-MATR3:YFP (Supplemental Table S1). Overall, our data find no obvious difference between WT and mutant MATR3:YFP expression in affecting global gene expression in this transient transfection paradigm in H4 neuroglioma cells.

**Table 1:** Genes influenced by over-expression of MATR3:YFP (no change in expression by YFP alone).

Gene Name	Log 2 fold change WT MATR3:YFP vs YFP	Log 2 fold change F115C MATR3:YFP vs YFP
***Genes induced by WT or F115C MATR3 Overexpression***		
**EGFR(↑)**	0.86	0.61
WNT5A	0.91	0.76
SPANXC	1.05	0.61
TNC	1.13	1.14
MIR711	1.64	1.35
AMTN	3.03	2.45
***Genes suppressed by WT or F115C MATR3 Overexpression***		
MMD	−0.87	−0.47
**CTSC(↓)**	−0.80	−0.39
**SEMA6B(↓)**	−0.90	−0.51
IFITM10	−0.98	−0.67
MXRA8	−1.04	−0.68
SEPP1(↓)*	−1.04	−0.66
ST8SIA4	−1.06	N.S.
EID1	−1.07	−0.86
PTPN13	−1.07	−0.64
**RAD51 AP1(↓)**	−1.08	−0.93
IGFBP3	−1.18	−0.67
**ENPP1(↓)**	−1.24	−0.64
PDE5A*	−1.27	−0.79
RGS2	−1.41	−1.44
**PCDH18(↑)**	−1.60	−1.05
PCDH20	−1.80	−0.96

Bold = Genes identified with altered expression by knockdown of MATR3 in^17^; arrows indicate whether the reported change in expression was up or down regulated.

*Genes identified with alternative splicing events regulated by MATR3.

With so few genes identified with altered expression, no clear clustering of functionality could be undertaken. With one exception, there were no obvious changes in the splicing of alternative exons for any of the 23 genes identified in Table 1 (examples of a subset shown Supplemental Figures S2–S11). The one exception was for the gene *CTSC*, which is alternatively spliced to produce mRNAs that encode a truncated protein (Supplemental Figure S12). The over-expression of WT or F115C MATR3:YFP appeared to suppress inclusion of two exons that generate a truncated mRNA and protein (Supplemental Figure S12). Interestingly, a TCTT motif is present immediately upstream of these exons and this motif was identified by Coelho and colleagues as a sequence commonly found upstream of exons repressed by MATR3^17^.

There were 59 coding genes that were induced in cells expressing YFP alone with either a greater degree (38 genes) or lesser degree (21 genes) of induction in cells expressing WT or F115C MATR3:YFP (Supplemental Table S2). Within this set of genes, there were 2 genes (*TMEM100* and *SERPINB2*) that were expressed at ≥100 RKPM and were identified as potentially being differentially regulated by WT and F115C MATR3 (Supplemental Table S1). However, because the expression of these genes was induced in cells expressing YFP alone it is unclear whether either of these genes is specifically regulated by MATR3. Interestingly, the list of genes that were induced by expression of YFP alone (Supplemental Table S2) was heavily populated by inflammatory modulators. There were 15 genes identified that were down-regulated by YFP expression with further down-regulation by expression of the MATR3:YFP fusion constructs. Whether these genes are candidates for regulation by MATR3 is unclear due to response of the gene to YFP expression alone. Although we identified only one non-coding RNA that showed altered expression, our study produced reads for multiple genes from nearly every class of non-coding RNA (Supplemental Table S3). Overall, the data show that the expression of relatively few genes was dysregulated by over-expression of WT or F115C MATR3 in the human neuroglioma cell line used for this study.

To determine whether MATR3 may auto-regulate its expression, we examined the levels of endogenous MATR3 mRNA levels by subtracting the reads for MATR3 coding exons that would be included in the cDNA gene for MATR3:YFP. The RPKM numbers for endogenous MATR3 in untransfected H4 neuroglioma cells were averaged 817 RPKM with an average of 568 RPKM for cells transfected with the vector for YFP alone (Supplemental Figure S13), yielding a 1.4-fold reduction in endogenous MATR3 mRNA levels (adj. p < 0.0055). There was no further reduction of endogenous MATR3 levels in cells expressing WT or F115C-MATR3:YFP (avg. RPKM 480 and 468 respectively; adj. p < 0.19 and 0.14, respectively, for YFP vs. WT and F115C-MATR3:YFP). Thus, we find no compelling evidence that MATR3 auto-regulates its expression in the H4 neuroglioma cells within the timeframe of these experiments.

### Interactome Analysis of WT and Mutant MATR3

To determine whether mutations in *MATR3* might disturb function by altering its normal interaction with binding partners, we constructed recombinant cDNA *MATR3* genes that encode C-terminal AviTag sequences that enable enzymatic biotinylation of the protein for affinity capture with avidin^25,26^. We generated WT-MATR3:AviTag, S85C-MATR3:AviTag, and F115C-MATR3:AviTag expression vectors and transfected these constructs into HEK 293 cells. HEK293 cells were selected to allow us to directly compare our data with a prior publication that used an antibody-based affinity capture method^18^. Similar to this prior study, we also used label-free spectra counting methods and statistical analyses to identify proteins that bind to each of the MATR3 variants. To control for background binding to affinity capture media, we incubated the avidin-magnetic beads with lysates of untransfected HEK293 cells. Data from five replicate control samples was compared to data from four replicates of samples from affinity capture with WT-MATR3-Avi, three replicates of capture with S85C-MATR3:AviTag, and three replicates of capture with S85C- or F115C-MATR3:AviTag. For the most robust binding proteins, the spectral count data were highly consistent (Table 2, Supplemental Tables S4 and S5). The criteria for identification as a MATR3 interacting protein was that the average fold change in peptide spectra between a MATR3:AviTag sample and control was ≥2 and p ≤ 0.05 by t-test, and that the number of spectra in the MATR3 samples was significantly over-represented by G-test^27,28^ in at least 2 experimental runs, and by SAINT score (≥0.90)^29^. By these criteria, we identified 123 proteins that were preferentially co-captured with WT-MATR3:AviTag (Supplemental Tables S4 and S5).

**Table 2:** Summary of spectral count data for the top 50 interacting proteins ranked by fold enrichment for MATR3:AviTag relative to controls.

	Control					WT-MATR3				S85C-MATR3			F115C-MATR3		
Experiment #	1A	2 A	3B	4B	5B	1 A	2 A	3B	4B	1 A	2 A	3B	1 A	2 A	3B
MATR3	6	9	2	5	5	134	133	717	515	170	171	217	134	147	773
**HNRNPM**	20	0	0	0	0	43	38	310	239	46	48	179	44	53	212
SFRS14	0	0	0	0	0	34	32	23	16	21	16	9	25	31	27
**PTBP3**	1	1	0	0	0	14	12	40	32	16	16	24	13	13	40
M0QYT0	0	0	0	0	4	21	17	24	33	24	21	19	22	26	58
**ADAR**	0	2	0	0	0	36	37	8	9	31	36	7	40	38	20
PRPF8	0	2	0	0	3	26	33	11	10	35	33	8	39	27	40
A0A024R8A7	4	6	0	0	0	39	36	43	37	35	41	20	39	45	45
**PTBP1**	4	6	0	0	4	26	22	96	65	35	44	48	29	40	199
**ZNF326**	0	0	0	0	0	19	19	22	13	23	15	4	20	22	19
PABPC1	3	4	0	0	0	10	17	38	35	15	8	22	17	17	34
**ILF3**	2	6	0	0	1	36	34	30	28	38	35	16	39	30	42
PABPC4(2)	0	2	0	0	0	13	17	22	19	12	7	13	12	10	14
SNRNP200	0	2	0	0	2	24	30	4	8	33	35	6	34	33	23
MYBBP1A(2)	0	0	0	0	1	22	21	10	12	25	22	5	25	27	43
B2R959	2	3	0	0	5	24	23	34	43	29	30	26	26	37	79
**HNRNPA2B1**	5	4	2	0	18	49	42	146	113	62	57	63	55	61	288
DHX30	0	0	0	0	0	23	24	5	8	18	15	1	23	18	8
IGF2BP3	1	0	0	0	0	17	18	13	10	16	17	2	17	14	18
BCLAF1	0	0	1	0	1	14	12	14	15	7	3	8	18	10	2
**HNRNPA3**	2	3	0	0	7	30	24	37	37	36	35	27	33	36	84
SYNCRIP	2	2	0	0	2	13	14	23	14	14	12	8	13	13	40
A0A024RC46	6	3	0	0	9	36	28	64	63	46	43	41	34	41	214
**HNRNPA1**	8	3	0	0	11	41	32	80	76	52	47	53	40	47	227
MOV10	0	0	0	0	0	18	24	6	4	17	16	1	23	30	11
ATAD3B	0	4	0	0	0	6	6	17	21	6	9	18	9	11	12
**HNRNPUL1**	1	0	0	0	0	18	15	12	5	15	12	4	16	20	16
THRAP3	0	0	0	0	0	13	13	12	10	13	13	8	17	12	3
DDX1	0	2	0	0	0	18	18	7	4	22	18	5	22	25	20
**HNRNPR**	2	2	0	0	4	17	17	24	15	20	19	9	21	19	46
RPL13A	0	1	2	0	1	7	9	13	15	9	7	10	9	9	20
RPL15	0	0	0	0	1	7	9	12	14	11	12	10	11	10	29
RPL6	3	6	0	0	1	16	16	29	22	14	18	19	18	20	43
GTPBP4	0	1	0	0	0	11	15	6	9	11	12	6	18	15	9
**HNRNPUL2**	0	0	0	0	0	14	13	6	8	11	12	1	15	18	11
NCOA5	0	0	0	0	0	16	13	11	1	26	20	4	15	15	12
RSL1D1	0	1	0	0	1	15	13	7	5	16	18	6	19	19	24
SMC1A	2	0	0	0	3	14	16	4	6	14	16	0	18	13	12
B0AZQ4	0	0	0	0	1	17	16	2	4	16	10	1	13	21	21
MYEF2	0	0	0	0	0	16	12	6	5	7	6	1	10	13	10
SMCHD1	0	0	0	0	2	12	20	3	4	18	14	4	30	20	11
IGF2BP1	6	8	0	0	3	35	31	30	36	34	26	15	30	35	51
EPPK1	0	0	6	0	5	28	33	15	9	35	35	7	47	27	19
**KHDRBS1**	3	3	0	0	2	13	11	18	19	14	14	16	15	12	30
DHX15	3	3	0	0	4	20	16	18	22	15	16	11	19	20	25
B3KY60	1	2	0	0	1	14	14	5	5	14	11	5	14	16	6
PRPF6	0	1	0	0	0	13	16	4	5	17	15	1	15	17	10
**ILF2**	1	4	0	0	4	15	11	23	18	14	18	8	17	19	20
LRPPRC	1	2	0	0	1	11	17	5	4	20	25	0	18	24	9

Experiment number indicates individual transfection and affinity capture experiments. The A and B designations indicate batches that were analyzed simultaneously. Bold Font indicates proteins that have been identified in other studies as interacting with MATR3; https://thebiogrid.org/115126, https://www.ebi.ac.uk/intact/, http://string-db.org,^19^. The shaded cells indicate instances in which the spectral counts for a particular protein were not statistically significant by G-test for that particular mass spectrometry run.

**Figure 1:**
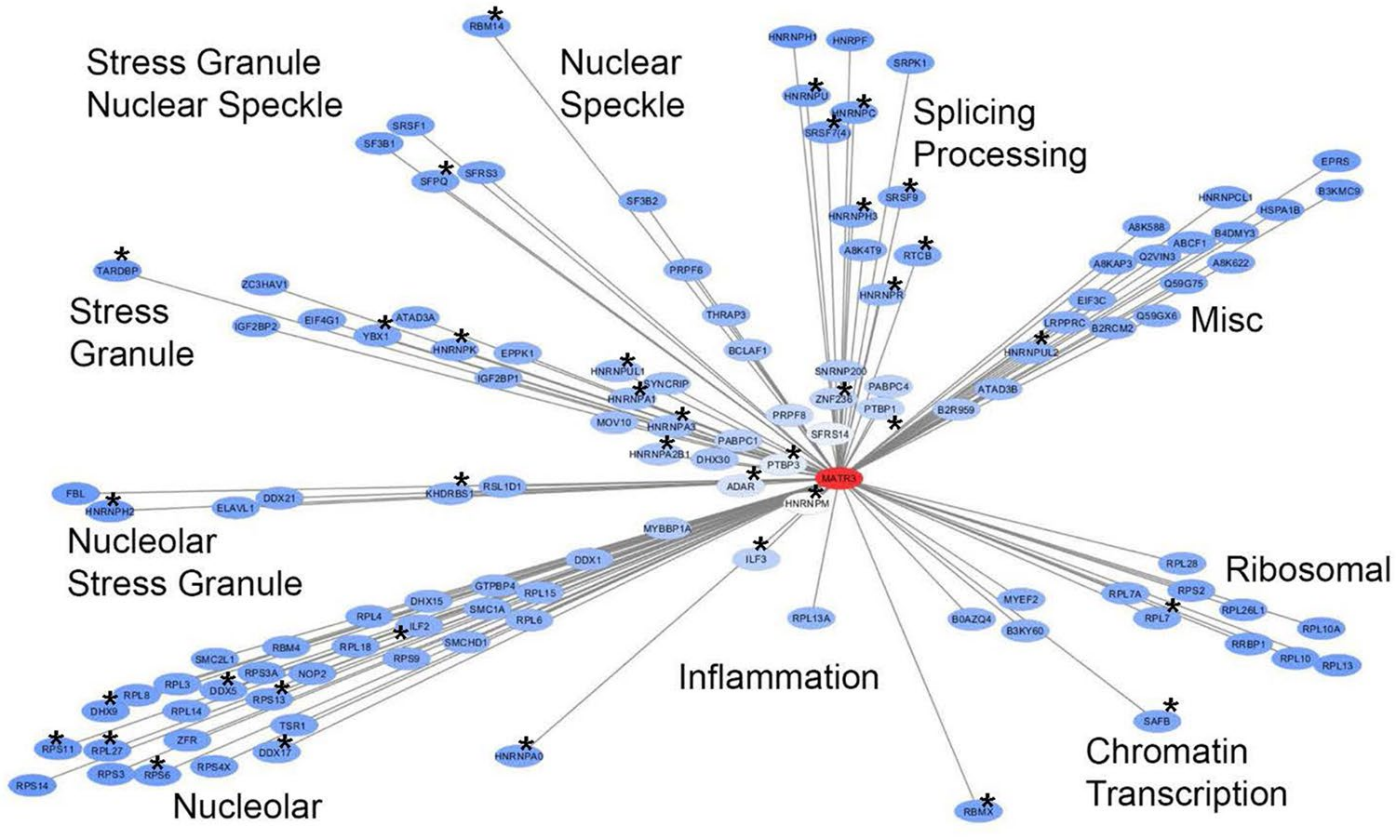
A graphic representation of the interactome for WT-MATR3 in human HEK293 cells. Proteomic mass spectrometry data from Supplemental Table S4 and the software program Cytoscape (version 3.5.1)^42^ were used to generate a graphic representation of MATR3 interaction. The intensity of shading of the oval containing the gene name is linked to the spectral counting data in Supplemental Table 4; the lighter the shading the higher the fold increase in spectral counts for a given protein relative to controls (untransfected cells; no expression of Avi-tagged MATR3). Proteins marked with an asterisk are interactors also identified by other studies (see Supplemental Table S4). The localization or function of the identified proteins was determined by three sources {www.uniprot.org} and^43,44^. Using quantification by label-free spectral counting, HNRNPM and numerous RNA processing factors including ADAR, PDBP3, SFRS14, and PTBP1 appear to be the strongest interactors with MATR3.

Many of the proteins we identified here have been reported by others to bind MATR3, using either affinity capture methods or yeast-2-hybrid methods (Fig. 1, marked by asterisk; Table 2, Bold Font; Supplemental Table S4, Bold Font, Red Font, and Green Highlight). The most abundant of these as determined by spectral count levels were HNRNPM, PTBP3, ADAR, PTBP1, ZNF236, ILF3, HNRNPA2B1, HNRNPA3, HNRNPA1, HNRNPUL1, HNRNPR, HNRPMUL2, KHDRBS1, PRPF6, and ILF2. The vast majority of the proteins identified as WT-MATR3 interactors were proteins identified as localizing to the nucleolus, proteins associated with stress granules or nuclear speckles, or proteins functioning in RNA processing and splicing (Fig. 1). More than half of the WT-MATR3-Avi interacting proteins were RNA binding proteins (possessing an RRM domain or other recognizable RNA binding motif), and more than half of these proteins possessed prion-like domains or regions of low structural complexity (≥50 aa in length) (Supplemental Table S4). Overall, these data strongly suggest that the primary class of proteins that strongly bind MATR3 are proteins known to be involved in RNA metabolism.

The interactomes for S85C-MATR3:AviTag or F115C-MATR3:AviTag were nearly identical to that of WT-MATR3:AviTag (Supplemental Table S5). The only difference noted was in analysis of data from cells expressing F115C-MATR3:AviTag, in which there were 9 proteins that showed higher numbers of spectral counts relative to controls (Supplemental Table S6). Overall, however, these findings indicate that disease-causing mutations do not cause dramatic changes in the interactome of MATR3.

To define whether interactions between MATR3 and its partners involved any of the recognizable sequence elements (RRM1, RRM2, ZnF1, and ZnF2), we obtained variants of MATR3, initially described by Salton *et al*.^13^, in which each RRM and ZnF motif were deleted. We used these constructs to generate novel AviTag constructs and then expressed them in HEK293 cells and performed affinity capture and proteomic analysis. For these experiments, we examined data from two replicates, using the same criteria of G-test for significance. In assessing the relative binding of these proteins to each of 4 deletion mutants, we normalized the spectral counts by a correction factor to account for different levels of each MATR3 deletion mutant that was captured (no correction needed for ΔRRM1 or ΔRRM2 mutants; correction factor for ΔZnF1 = 1.2 and for ΔZnF2 = 2). Deletion of RRM1 caused significant reductions in the binding of 19 proteins (Supplemental Table S4). MYBBP1A, SYNCRIP, A0A24RC46, HNRNPR, ILF2, RTCB, DDX21, RPL3, RPS11 and FBL were significantly reduced by RRM1 deletion with no trend of >2-fold for any other deletion mutant. PRPF8, SNRNP200, GTPBP4, SMCHD1, DHX15, PRPF6, A8KAP3, DHX9 and EPRS were significantly reduced by RRM1 or ZnF1 deletion with no trend of >2-fold for any other deletion mutant (Supplemental Table S4). Only one protein showed a significant, and selective, reduction in binding with deletion of RRM2 (HNRNPM). Remarkably, 37 of the proteins identified as interacting with WT-MATR3 showed increased binding with deletion of the RRM2 domain (Supplemental Table S4). Additionally, in cells expressing the WT-MATR3:AviTagΔRRM2 construct we identified 142 proteins that specifically co-purified (Supplemental Table S7). Only three proteins were significantly, and selectively, diminished in MATR3 binding by deletion of ZnF1 (ILF3, IGF2BP1, EPPK1), with no proteins showing a selective reduction by ZnF2 deletion (Supplemental Table S4). Thus, although deletion of RRM1 and, or, ZnF1 reduced the binding of a subset of interacting partners for MATR3, the most dramatic effect observed was by deletion of RRM2 which caused the aberrant binding of over 140 proteins.

### Analysis of WT and Mutant MATR3 Subcellular Localization

**Figure 2:**
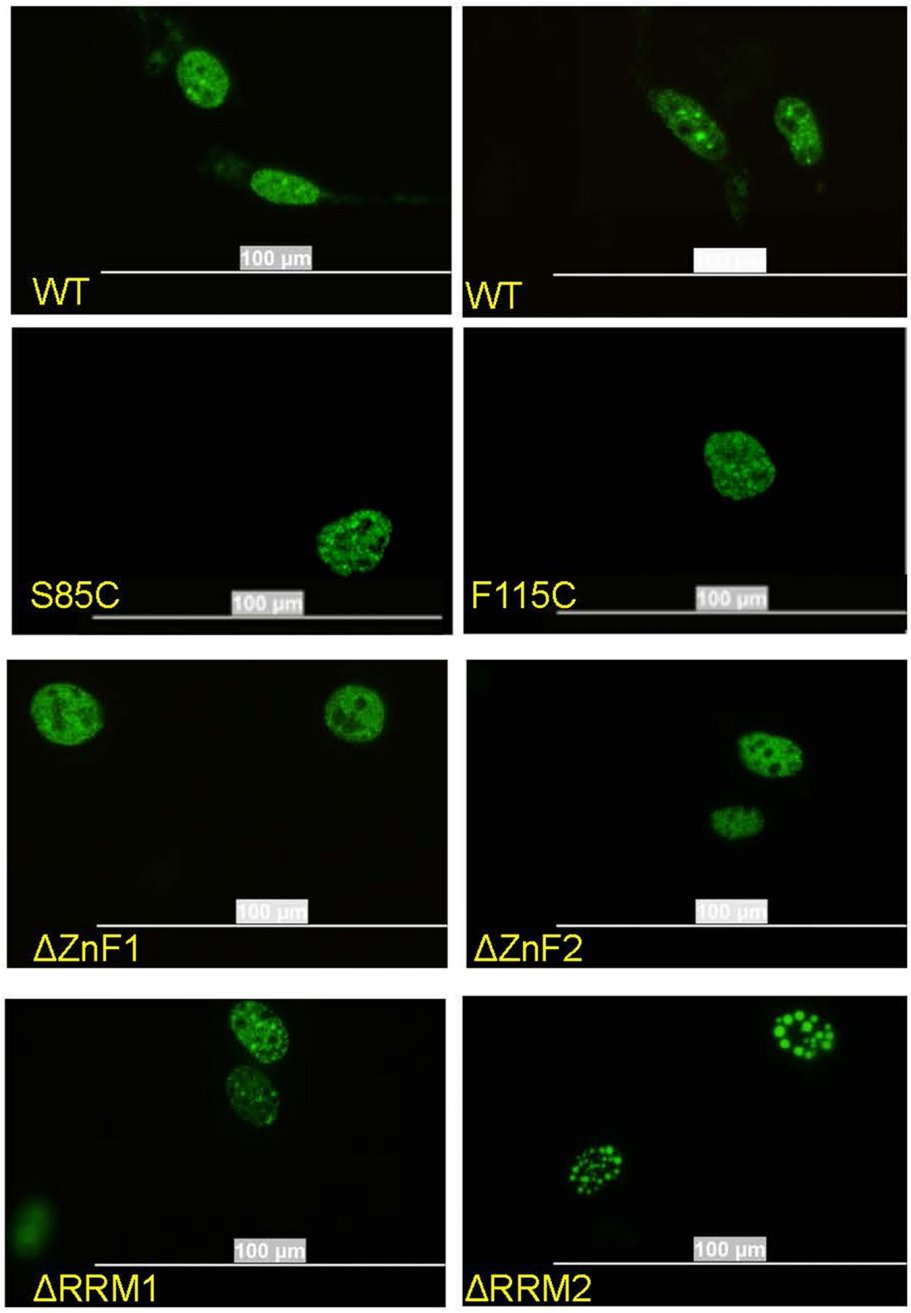
Distribution of MATR3:YFP in nuclear compartments of C2C12 cells. Mouse C2C12 cells were grown on glass cover slips and transiently transfected with pEF.Bos vectors encoding MATR3:YFP variants noted on the figure. At 24 hours post-transfection, the cells were fixed and imaged. The images shown are representative of images seen in 3 independent transfections, viewing 30 to 50 cells on each cover slip (data quantified in Table 3). WT MATR3 and MATR3 lacking ZnF1, ZnF2, or RRM1 sequences show a predominantly diffuse distribution within the nucleus with variable levels of small puncta. MATR3 lacking RRM2 organizes into large uniformly spherical structures. Scale bar = 100 µm.

In prior studies, using multiple cell lines, WT and mutant MATR3 expressed as either untagged proteins, or as fusion proteins with YFP, were observed to be primarily localized to the nucleus where they displayed a diffuse distribution with variable levels of discernable puncta^4^. Although our proteomics data suggest that MATR3 might be present in nucleoli, we observed no obvious localization of the protein in nucleoli, which appeared as essentially empty holes in the nucleus (Fig. 2)^4^. To relate our proteomics data to any changes in MATR3 distribution in the cells, we examined a panel of MATR3 mutants, fused to YFP, in which each Zn finger domain (ZnF) or RRM domain was deleted. For these studies, we used mouse C2C12 myoblast cells, which represent a relevant cell type for myopathy associated with MATR3 mutations. More importantly, these cells possess a morphology that facilitates microscopic studies (the H4 cells used in RNA-seq studies were highly mobile and the HEK293 cells used in the proteomic studies tended to clump and adhered poorly to glass cover slips). We observed a diffuse distribution of WT-, S85C, or F115C-MATR3:YFP with puncta within the nucleus (Fig. 2) – in agreement with our previous study in other cell types including human H4 neuroglioma cells^4^. MATR3:YFP with deletions of either ZnF1 or ZnF2 produced patterns that were similar to WT MATR3:YFP (Fig. 2). Most cells expressing MATR3:YFP with deletion of RRM1 appeared similar to WT MATR3:YFP, but in cells expressing MATR3:YFP with deletion of RRM2, the protein appeared to be coalesced into spherical droplets (Fig. 2). To quantify these phenotypes, a blinded observer (R.D.) examined fields of images of cells expressing each variant taken from 3 replicate transfection experiments and categorized the appearance of the MATR3:YFP as Phenotype I (WT-like), Phenotype II (droplets), or Phenotype II + (large droplets). In cells transfected with each construct, we observed consistent phenotypes (Table 3), with the only construct showing organization of MATR3:YFP into spherical structures being the variant lacking RRM2 (MATR3:YFPΔRRM2). In this case, about 50% of the cells expressing the fusion protein produced spheres, with a subset of these producing large structures (Table 3). The spherical structures formed in cells expressing MATR3:YFPΔRRM2 were weakly stained by Nile Red (Supplemental Figure S14), a dye that specifically stains intracellular lipid droplets^30^. Introduction of the F115C mutation into MATR3:YFPΔRRM2 did not obviously alter the formation of the spherical structures, but introduction of the S85C mutation largely inhibited the formation of the spheres (Supplemental Figure S15).

**Table 3:** Quantification of spherical structures formation by MATR3:YFP constructs.

Construct transfected into C2C12 cells	Total # cells YFP positive	# cells w/o droplets	# cells with spheres	# cells with large spheres	% cells with spheres
MATR3:YFP-WT	373	373	0	0	0
MATR3:YFP-ΔRRM1	318	318	0	0	0
MATR3:YFP-ΔRRM2	197	107	77	13	47%
MATR3:YFP-ZnF1	182	182	0	0	0
MATR3:YFP-ZnF2	80	80	0	0	0
MATR3:YFP-S85C	167	167	0	0	0
MATR3:YFP-S85C- ΔRRM2	196	141	55*	0	28%
MATR3:YFP-F115C	138	138	0	0	0
MATR3:YFP-F115C- ΔRRM2	101	17	81	3	83%

*The spherical structures that formed tended to be smaller than either WT or F115C MATR3:YFP

**Figure 3:**
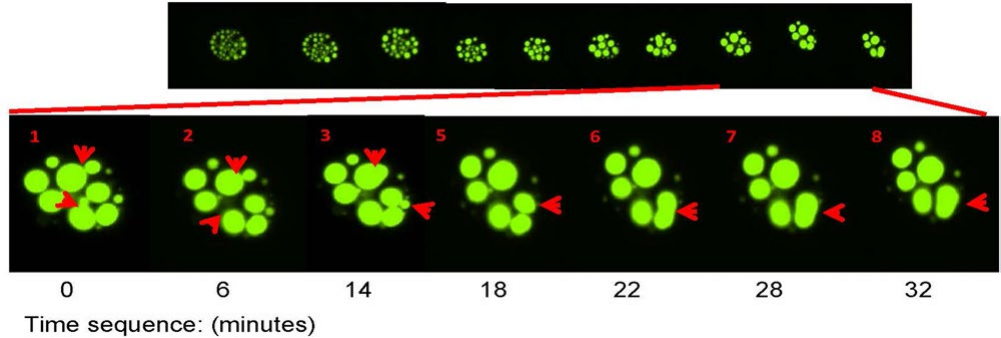
Images from time-lapse video demonstrates fusion of MATR3:YFPΔRRM2 spheres in C2C12 cells. An example of the timing of fusion is illustrated in this panel of images. Two independent fusion events can clearly be seen to occur over a period of 15–30 minutes in each case. The example shown is representative of observations made in 3 separate transfection experiments tracking 20–30 cells in each field of view. Original magnification 20X.

To examine further the basis for the morphology of MATR3:YFPΔRRM2, we used time-lapse imaging of live cells, capturing images every 30 seconds over a 24-hour period. In analyzing these images, we observed abundant small spherical structures ranging from 1 to 3 µm that gradually fused to produce larger spheres (>10 µm in diameter; Fig. 3, Video S1). The fusion of these spheres took place over varied intervals, ranging from a few minutes (Fig. 3) to several hours (not shown). Interestingly, analysis of time-lapse video of cells expressing MATR3:YFPΔRRM1 revealed that this mutant also produced spheres, but the structures were not as stable as those formed by the RRM2 deletion mutant (data not shown).

**Figure 4:**
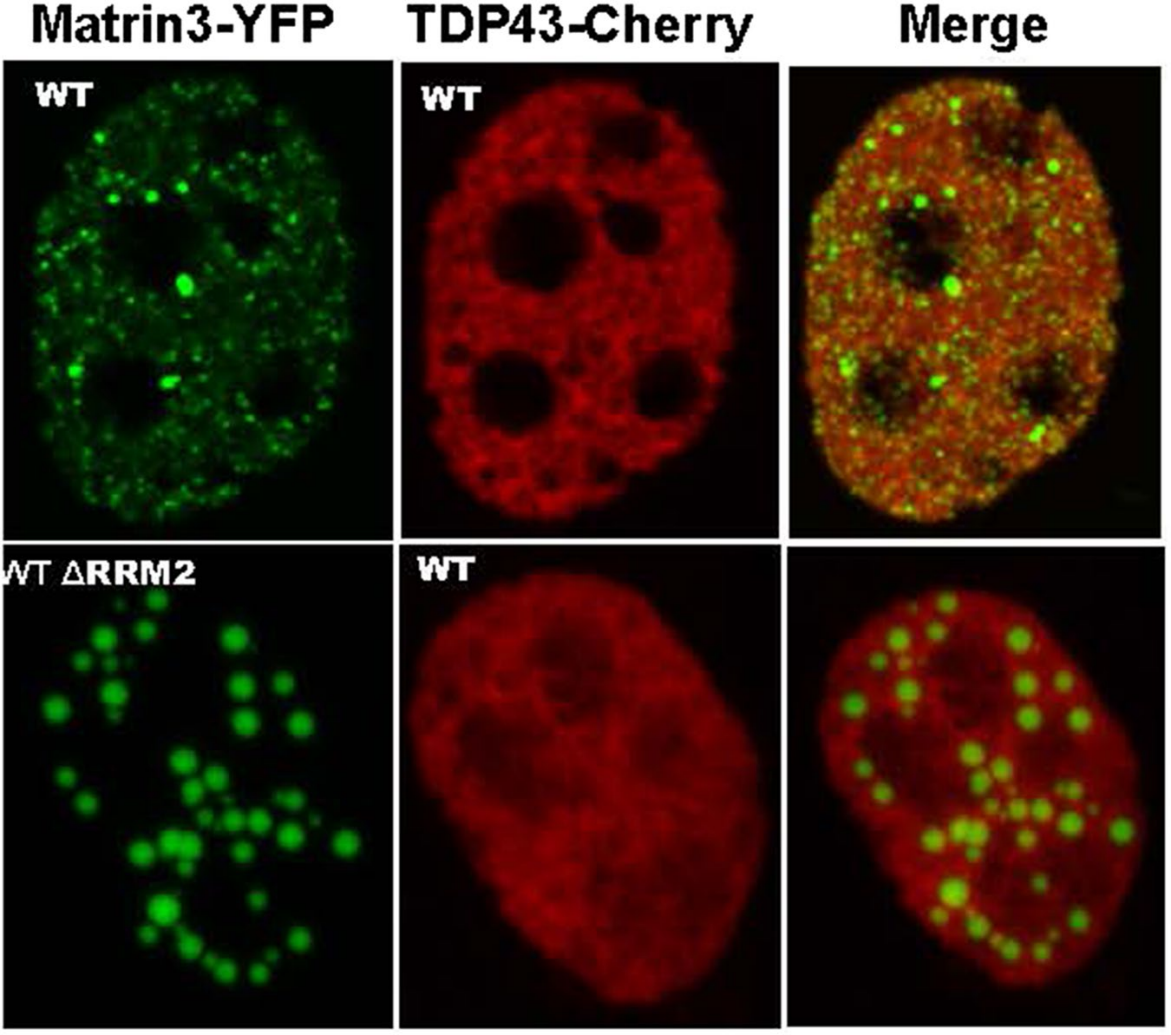
Lack of WT-MATR3:YFP and WT-MATR3:YFP ΔRRM2co-localization with TDP-43:mCherry in C2C12 cells. Mouse C2C12 cells were grown on glass cover slips and transiently transfected with pEF.Bos vectors encoding MATR3:YFP variants noted on the figure. At 24 hours post-transfection, the cells were fixed and imaged. The images shown are representative of what was found in at least two separate transfection experiments analyzing 50–100 cells in each experiment (original magnification 60X with 2X digital enlargement). Each image is from a single z-plane captured on a confocal microscope (see Methods). WT-MATR3:YFP and WT-TDP-43:mCherry show limited overlap, for example TDP-43:mCherry is not seen in puncta of WT-MATR3:YFP or the spherical structures formed by MATR3:YFPΔRRM2.

Previous studies have reported that MATR3 can interact with TDP-43^3^. In our proteomic study, we similarly detected an interaction between TDP-43 and MATR3, but the strength of interaction was among the weakest and we did not observe that either the S85C or F115C mutations strengthened the interaction (Supplemental Table S4). The interaction of MATR3:AviTagΔRRM2 with TDP-43 was also relatively weak (Supplemental Table S4). In cells co-expressing WT MATR3:YFP and WT TDP-43:mCherry, we observed limited co-localization (Fig. 4). Similarly, we observed minimal co-localization WT TDP-43:mCherry in spheres formed by MATR3:YFPΔRRM2 (Fig. 4). These findings largely confirm our proteomic data.

**Figure 5:**
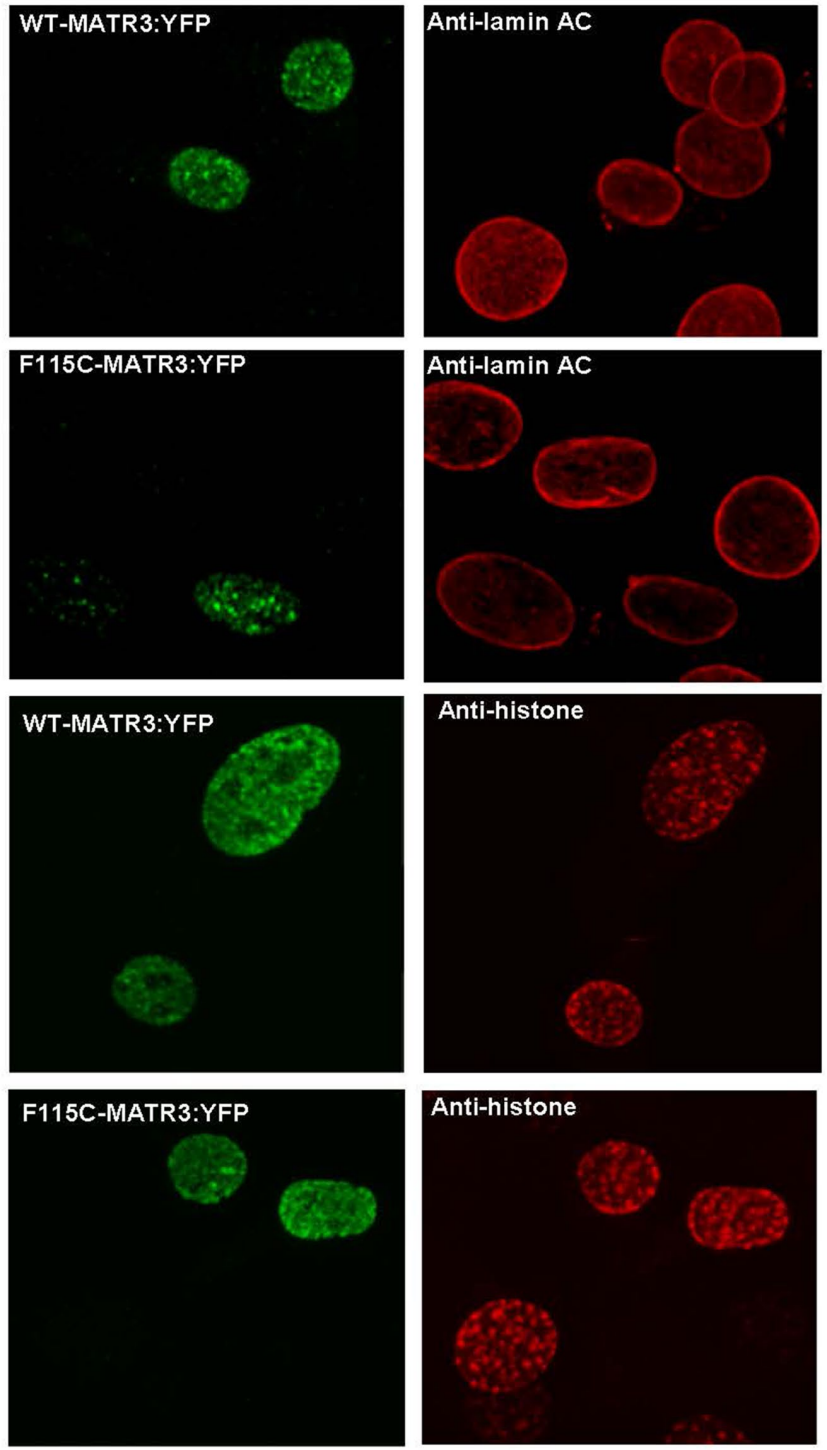
Minimal co-localization of MATR3 with lamin A/C or histones. Mouse C2C12 cells were grown on glass cover slips and transiently transfected with pEF.Bos vectors encoding MATR3:YFP variants noted on the figure. At 24 hours post-transfection, the cells were fixed, immunostained with antibodies to lamin A/C (Sigma-Aldrich #SAB4200236) or histone H3 (Abcam #ab8898) and imaged. The images shown are representative of images seen in 3 independent transfections, viewing 30 to 50 cells on each cover slip. Each image is from a single z-plane captured on a confocal microscope (see Methods). Nikon elements software was used to determine the extent of interaction between MATR3 and lamin A/C or histone H3 (Supplemental Figures S16 and S17). MATR3 shows limited co-localization with either lamin A/C or histone H3.

Our proteomics data strongly indicate that the primary interactors for MATR3 are proteins involved in RNA processing and splicing. We found limited interactions with proteins associated with chromatin or the nuclear matrix. Immunofluorescence co-localization studies of MATR3 with nuclear lamin and histones confirmed our proteomic data (Fig. 5). Nikon elements software was used to determine the extent of interaction between MATR3:YFP and immunolabeled lamin A/C or histone. The software reported little or no interaction between MATR3 and lamin A/C at the nuclear membrane (Supplemental Figure S16). Within the nucleoplasm, the levels of lamin A/C were very low as compared to the expressed MATR3:YFP and it was difficult to determine whether that was some level of co-localization (Supplemental Figure S17). The same analysis for MATR3 and histone co-localization suggest that a small fraction of MATR3 may be in close proximity to histones (Supplemental Figure S17). Overall, our studies of MATR3 localization in nuclei was consistent with our proteomic interaction network findings. WT and mutant MATR3 appear to be primarily localized to the nucleoplasm where the protein could interact with multiple factors involved in RNA processing.

## Discussion

To begin to define the impact of disease-causing mutations in MATR3 on the ability of MATR3 to regulate gene expression and interact with binding partners, we have used RNA deep sequencing analysis, affinity capture proteomics, and microscopy to examine WT and mutant MATR3 expressed in cultured cells. The over-expression of WT or mutant MATR3 produced relatively few changes in gene expression that met criteria of occurring only in cells over-expressing the MATR3 constructs, and producing changes in levels of ≥1.6 fold for genes with ≥100 RPKM. There were no obvious examples of genes that were specifically dysregulated by the over-expression of F115C-MATR3 within these criteria. To determine whether disease-causing mutations in MATR3 may disrupt its normal interaction network, we conducted affinity-capture proteomic studies in HEK293 cells to identify proteins interacting with WT, S85C, and F115C MATR3. The major classes of proteins in the MATR3 interactome of HEK293 cells were nucleolar proteins, proteins associated with stress granules, and proteins involved in RNA splicing/processing. Thirty-nine of the proteins identified in our study had been detected as interactors with WT-MATR3 in other studies and thus can be categorized as interacting partners with high confidence (see Fig. 1, marked by asterisk, and Supplemental Table S4, marked by Bold or Red Font, and green highlight). Within the limitations of using spectral counting to assess strength of interaction, and focusing on the partners that had been replicated by others, there were no obvious differences between the interactome of WT and either S85C or F115C-MATR3. Overall, our data indicate that disease-causing mutations do not severely affect the binding of MATR3 to its normal partners. Our interactome study found little evidence of MATR3 interaction with nuclear lamins or components of chromatin, and our microscopy studies yielded similar data. Our interactome data indicated that MATR3 only weakly interacts with TDP-43, a finding confirmed by co-localization microscopy. Additionally, no major differences in the subnuclear location of WT and mutant MATR3 were noted. Under the conditions used here, our study indicates that disease-causing mutations in MATR3 do not produce changes in the protein that manifest by dysregulating gene expression or protein interaction networks when over-expressed in culture cell models.

We compared our RNA-seq data for over-expression of MATR3 to a study of gene expression in HEK cells in which endogenous MATR3 had been knocked down^17^. This study reported that reducing MATR3 levels caused ≥2-fold changes in the expression of only 61 genes. There were seven genes from our list in Table 1 that were also identified in the Coelho *et al*. study as regulated by MATR3; *EGFR*, *CTSC*, *SEMA6B*, *SEPP1*, *RAD51AP1*, *ENPP1*, and *PCDH18*. Two of the genes in Table 1, *SEPP1* and *PDE5A*, were identified by Coelho *et al*. as producing mRNA whose splicing is regulated by MATR3. In our cell models, we did not observe that over-expression of MATR3:YFP produced an obvious alteration in the splicing of either of these genes (see Supplemental Figures S3 and S5). As indicated above, with one potential exception (*CTSC*), we did not observe obvious shifts in splicing for any of the genes identified in Table 1 as regulated by MATR3:YFP over-expression (see Supplemental Figures S2–S11 for examples). Most importantly for this study, we could find no compelling evidence that the F115C variant of MATR3 differed from WT MATR3 in regulating gene expression.

Our study of the MATR3 interactome in HEK293 cells indicates that MATR3 binds multiple nucleolar proteins, proteins associated with stress granules, and proteins involved in RNA splicing/processing. The lack of evidence in our data set for interaction with proteins of the TREX complex as recently described^19^ may reflect differences in the cell models used, or the method of MATR3 expression (stable versus transient over-expression). A major consideration in interpreting these interactome data is that the proteins identified as interacting with MATR3 are themselves often in complex with other proteins to form macromolecular structures. Identifying which interactions are direct binding of MATR3 to the target protein is difficult. One way to view the data is that associations that are identified in multiple studies, using different approaches are more likely to be direct protein:protein interactions. Across all of the interaction data sets on MATR3, the proteins that have been most consistently reported to interact with MATR3 are proteins known to be involved in RNA processing or have been associated with RNA stress granules (see Fig. 1). It is somewhat surprising that a subset of nucleolar proteins are among those that are consistently found as MATR3 binders. The observed interaction with nucleolar proteins in these data must reflect events that occur outside of the nucleolus itself since we have not observed tagged or untagged MATR3 within the nucleolus. Many nucleolar proteins are multifunctional, and thus could be interacting with MATR3 or complexing with other MATR3 binding proteins outside of the nucleolus. Additionally, many of the binding events ascribed to nucleolar proteins involved relatively weak interactions with ribosomal proteins, and it is possible that these interactions occur as a consequence of over-expression. Some of the observed interactions with proteins involved in RNA splicing/processing could be due to RNA tethering (different RNA binding proteins in the same RNA) or otherwise RNA-dependent associations. Deletion of RRM1 caused significant reductions in the binding of 19 proteins with another 9 proteins showing reduced binding when RRM1 or ZnF1 were deleted. Only HNRNPM showed a significant, but only partial, reduction in binding with deletion of RRM2. Notably, there were relatively few proteins in which binding to MATR3 was abrogated by deletion of RRM1 or RRM2, suggesting that most of the observed interactions may be direct protein:protein interactions, or interactions of MATR3 with a complex of proteins.

Although we observed no significant differences in the profile of proteins binding to WT and S85C MATR3:AviTag, for F115C-MATR3 there were 9 proteins that could be more strongly associated (Supplemental Table S6). However, all but one of these 9 proteins were identified here for the first time and thus will require additional verification. Because we did not observe increased binding of these 9 proteins to S85C-MATR3:AviTag, whether these may be disease relevant changes in protein:protein interaction is unclear. Overall, the data can be viewed as indicating that mutant MATR3 is likely to be able to interact with most of its normal binding partners and that there could be subtle alterations in binding affinity that may be worthy of examination more carefully in other model systems expressing mutant MATR3 at physiologic levels.

As mentioned above, several studies have reported that MATR3 interacts with TDP43^3,20^. Although we find interactions between WT or mutant MATR3 and TDP-43, these interactions were relatively weak in the HEK cell model used here. It is possible that post-translational modifications to either of these proteins that occur specifically in neuronal cells could modulate this interaction to be more robust than observed here. A recent study has reported that WT MATR3 immunoreactivity is associated with a subset of TDP-43 immunoreactive inclusions in sporadic ALS patients^5^, suggesting that there may be pathological settings in which these proteins interact more strongly.

Remarkably, the binding of 36 proteins that normally bind WT-MATR3 was selectively increased by the deletion of RRM2 (see Supplemental Table S4, red highlight), with an additional 142 proteins that were selectively bound by the RRM2 deletion mutant (see Supplemental Table S5). The increased binding of proteins by the RRM2 deletion mutant was accompanied by a dramatic alteration in the nuclear distribution of the protein to form spherical structures. The majority of proteins identified as interacting with WT-MATR3 possessed prion-like domains (PrLDs) or sequence elements predicted to be disordered (see Supplemental Table S4). The predominance of these proteins as interactors for MATR3 may be a reflection of the predominance of these types of proteins in nuclear compartments. Many proteins that are found in stable intranuclear proteinaceous membrane-less organelles are characterized as possessing sequence motifs that are intrinsically disordered^31^. One characteristic of proteins that are found in these structures is the ability to undergo liquid phase transition *in vitro*, forming liquid droplet-like structures^32–34^. The spherical structures formed by MATR3:YFPΔRRM2 resemble the liquid-like droplet structures formed by TDP-43 and FUS *in vitro*^32,35,36^. A hallmark of liquid-like structures is the ability to fuse into larger “droplets” as we have observed here for MATR3:YFPΔRRM2. Although MATR3 lacks a recognizable PrLD, large portions of its sequence upstream of RRM1 (aa 1–397) and downstream of RRM2 (aa 572–847) have been described as intrinsically disordered^17^. Segments of low complexity, including but not limited to PrLD elements, appear to be the primary determinant in whether a protein can undergo liquid-liquid phase transition^32–34^. Whether MATR3:YFPΔRRM2 is itself undergoing phase transition to produce liquid-like droplets or is associating with other nuclear proteins in such structures will require further study. Our interaction data on MATR3:AviTag YFPΔRRM2 could be an indication that this mutant is aberrantly associating with other proteins that are already in such liquid-like structures since more than 50% of the novel interactions with the ΔRMM2 mutant are with proteins that possess low complexity domains. Another possibility is that the deletion of the RRM domain has liberated the protein from association with RNA, allowing it to more freely self-associate into these spherical structures. Our interaction data on WT-MATR3:AviTag are consistent with the idea that MATR3 may be prone to interact with proteins in liquid-like structures of the nucleus given that a majority of the binding partners we identify for the protein possess PrL domains or other low complexity sequence elements (see Supplemental Table S4).

In summary, we have conducted an initial characterization of the impact of disease-causing mutations in MATR3 on the biology of the protein in relation to functions in gene regulation and RNA processing, in relation to its normal interaction network, and in relation to its normal localization in the cell. We find that the F115C mutation has little obvious impact on the gene regulation function of MATR3 and that the S85C and F115C mutations have minimal impact on the interactome of MATR3. In the course of our analysis, we observed that the RRM2 motif of MATR3 is a major modulator of the intranuclear distribution of MATR3 with deletion of this motif causing the protein to exhibit an altered distribution into spherical structures. Interestingly, loss of the RRM2 domain induced a large number of apparently aberrant protein:protein interactions. Although our RNA-seq data revealed limited changes in RNA splicing when MATR3 is over-expressed, our interactome data are consistent with other prior studies that suggest MATR3 primarily functions in regulating RNA processing. An unequivocal finding of our study is that RRM2 motif is critical in regulating protein:protein interactions and intranuclear localization of MATR3.

## Materials and Methods

### Cloning of vector plasmids

*MATR3* cDNA was obtained from Thermo Scientific (catalog number MHS6278-202757255; Waltham, MA, USA). PCR was performed using this cDNA as template with engineered primers that enabled cloning into a derivative the of pEF-BOS vector^37^. Yellow fluorescent protein (YFP) was fused to the C-terminus of MATR3 by PCR using the pEF-BOS.MATR3 vectors as templates, with forward and reverse primers that enabled insertion into the vector, using the Infusion HD kit from Clontech (catalog number 639649, Mountain View, CA). Flag- *MATR3* delZnF1 (plasmid #32884), Flag- *MATR3* delZnF1 (plasmid #32883), Flag- *MATR3* del RRM1 (plasmid #32881), Flag- *MATR3* delRRM2 (plasmid #32882) vectors was obtained commercially from Addgene (Cambridge, MA, USA). cDNAs inserts from these plasmids amplified by PCR, excluding the Flag tag, and were cloned into a pEF-BOS.MATR3:YFP vector by swapping out MATR3 cDNA sequences, using the Infusion HD kit. BOS-MATR3:YFPΔRRM2-S85C and F115C were generated by site directed mutagenesis with a Quickchange II XL kit (Agilent Technologies, cat# 200521, Santa Clara, CA). BOS-TDP-43-mCherry was produced by PCR amplification of the fusion coding sequences in CTR0-TDP-43-mCherry (Addgene plasmid #28206), followed by insertion into the pEF.BOS vector using an Infusion HD kit (Clontech/Takara Bio USA, Mountain View, CA). All constructs were verified by DNA sequencing before use with additional sequencing of plasmid DNA preparations to guard against any potential labeling errors.

### RNA-seq Studies

H4 neuroglioma cells were transfected with pEF.BOS vectors for YFP, WT-MATR3:YFP, and F115C-MATR3:YFP using Lipofectamine (Invitrogen, Cat # 15338-100, Carlsbad, CA). After 24 hours, the cells were sorted in a fluorescence activated cell sorter (Interdisciplinary Center for Biotechnology Research at the University of Florida). RNA from the isolated fluorescent cells was extracted using Trizol (following the manufacturer’s protocol; ThermoFisher Scientific, cat# 15596026). Total RNA was used to build barcoded TruSeq Stranded mRNA libraries for paired-end sequencing runs by the NextGen DNA sequencing laboratory of the University of Florida Interdisciplinary Center for Biotechnology Research. Illumina RNA-seq reads were de-multiplexed based on sample-specific barcodes using fastq-tools yielding ~30 million reads per sample. Reads were mapped to the human genome (hg19) using OLego^38^ and bedgraph files were generated using BEDtools to facilitate visualization of read coverage on the UCSC genome browser. Quantification of reads associated with annotated genomic features was performed using Quantas^39^ followed by normalization and determination of statistically reproducible changes using edgeR^40^. To identify differentially expressed genes, we filtered the data to identify genes in which the average RPKM reached a threshold of |log_2_ fold change| ≥1 and the adjusted p ≤ 0.01 for comparison of 3 replicates of cells transfected with either WT-MATR3:YFP or YFP alone (n = 3 for each construct) to 3 replicates of untransfected cells. Supplemental Table S1 lists all of the expressed genes ranked by log fold change (log FC) for the aggregate data comparing 3 data sets for untransfected cells to 3 data sets for cells expressing either YFP alone or WT-MATR3:YFP. To distinguish between the effects of DNA transfection and gene expression from any specific effects of WT or F115C MATR3:YFP expression, we compared the data sets for 3 replicates of cells expressing YFP alone to 3 replicates each of cells expressing WT or F115C-MATR3:YFP. In these comparisons we again sought to identify genes that reached a threshold of |log_2_ fold change| ≥1, but lowered the threshold for adjusted p value to p ≤ 0.05. Supplemental Table S1 also provides lists of the genes identified as differentially expressed in comparing the cells expressing YFP alone to cells expressing WT or F115C-MATR3:YFP. The genes are listed in rank order of logFC for the aggregate data sets for each condition. Notes are provided to indicate the maximum number of reads for each gene.

### Proteomic Studies

HEK 293 cells were transfected with wild-type and mutated Avi-tagged plasmids, using Lipofectamine 2000 (Invitrogen, catalog #11668-019, Carlsbad, CA) according to the manufacturer’s protocol. Twenty-four hours post transfection, the cells were rinsed three times with 1x PBS and then harvested by scraping. The cells were spun at 3000 xg for 5 min and the supernatant was discarded. The cells were resuspended in 50 mM Tris, pH 7.4, with 1% NP-40 and Protease Inhibitor Cocktail (Sigma Aldrich #P8340). The cells were lysed by sonication (10 sec, 3 times) and spun 5 min at 3000 xg, retaining the supernatant. To pre-clear endogenous biotinylated proteins, the cell lysate was incubated with Dynabeads MyOne Streptavidin T1 (Life Tech #65601) for 30 min on a Nutator. Using a magnet to separate the beads, the supernatant was transferred to a new tube and incubated for 2 h at 30 °C with biotin protein ligase (Genecopoeia #BIRA500). Samples were purified through PD SpinTrap G25 columns (Fisher #45001527) and once again incubated with Streptavidin beads for 30 min. The beads were separated with a magnet and the supernatant was discarded. Beads were washed with 1% NP-40/50 mM Tris, pH 7.4, for 10 min on the Nutator. The supernatant was removed and the beads were resuspended in 1% NP-40/50Mm Tris, pH 7.4, and transferred to a new tube. The beads were washed 3 more times and then suspended in 25 µL 1x Laemmli buffer.

The samples in Laemmli buffer were boiled (5 min, 95 °C) and the condensate was spun down. The Dynabeads were separated with a magnet and 2 µL of each sample was electrophoresed on an 18% Tris-Glycine gel. After electrophoresis, the proteins were transferred to a nitrocellulose membrane and probed with a rabbit anti-MATR3 antibody to validate expression of the tagged proteins. The remaining sample was loaded onto a 4–20% Tris-Glycine gel and electrophoresed for 15 min at 125 V in which time the proteins migrated about 1 cm into the gel. The gels were stained with Coomassie blue in 7% acetic acid with 40% methanol for 2 h. The gel was destained overnight in 7% acetic acid and 40% methanol. The total protein in each lane, contained within 1 cm of gel, was excised by cutting the gel with a clean scalpel and then further dicing each gel slice into 1 mm^3^ pieces. After incubation in water for 30 min, the gel pieces were washed 3 times in 50% acetonitrile with 50 mM ammonium bicarbonate for 15 min. The samples were dried down in a speedvac for 15 min before reduction with 45 nM of dithiothreitol for 30 min at 55 °C and alkylation in 100 mM iodoacetamide (30 min, in the dark). The gel pieces were then washed in 50% acetonitrile with 50 Mm ammonium bicarbonate for 15 min, 3 times. The gel pieces were completely dried in a speedvac and placed on ice and incubated with 12.5 ng/µL of trypsin (Sigma T6567-5 × 20UG) in 50 mM ammonium bicarbonate for 1 h and then placed in a 37 °C incubator overnight (~16 h). The reaction was stopped with 5% formic acid in 50% acetonitrile (30 min incubation). The digested peptides were extracted in 50% acetonitrile and then 100% acetonitrile. Peptides were dried down in a speedvac.

The dried samples were suspended in 0.1% trifluoroacetic acid (TFA). The Zip-Tip was washed 3 times with acetonitrile and 3 times with 0.1% TFA. Next, the sample was slowly pipetted up and down the Zip-Tip five times and discarded. The Zip-Tip was washed 3 times with 0.1% TFA and the peptides were eluted in 80% acetonitrile with 0.1% TFA into a clean tube, pipetting up and down 5 times. Samples were dried down in a speedvac. The digested peptides were analyzed with a Q-Exactive (Thermo Fisher) Mass Spectrometer. Each sample was initially resuspended with a 0.1% formic acid in 100% water solvent. Then was loaded through an Acclaim Pepmap 100 pre-column (20 mm × 75 mm; 2 mm-C18) and a Pepmap RSLC analytical column (250 mm × 75 mm; 2 μm-C18) at a 300 nl/minute flow rate during a linear gradient from solvent A (0.1% formic acid (v/v)) to 40% solvent B (0.1% formic acid (v/v), 80% acetonitrile (v/v)) for 90 minutes, followed by ramping up to 98% solvent B for additional 30 minutes. All MS/MS samples were analyzed using Mascot (Version 2.40). Scaffold Proteome Software (Version 4.8.2, Portland, OR, USA) was used to validate MS/MS-based peptide and protein identifications. Peptide identifications were accepted if they could be established at >95% probability as specified by the Peptide Prophet algorithm. Protein identifications were accepted if they were identified by at least two peptides and could be established at >99% probability using the Peptide Prophet algorithm.

Spectral counting was used to determine the relative abundance of proteins in the samples following published protocols^27,41^, in which the change in abundance was determined by the ratio of the total number of identified MS/MS spectra for a particular protein between the wild-type MATR3 and mutated MATR3 samples. A statistical G-test was utilized to determine the statistical probability that the abundance of a particular protein in a MATR3 affinity capture sample was significantly greater than chance (p < 0.05). To increase the statistical power of the G-test analysis and overall spectral counting sensitivity of proteins identified with high confidence (99% protein confidence, 95% peptide confidence, and containing two unique peptides), we included data sets of peptides with lower Mascot scores that represent positive identifications at 50% probability to match. To further establish whether a protein was over-represented in fractions bound to Avi-tagged MATR3, we also analyzed the data by T-test and SAINT score^29^. The SAINT algorithm uses regression analysis to determine the probability that a protein identified in the MATR3-binding fraction was not a consequence of random chance.

### Cell lines, transfection and immunohistochemistry

C2C12 mouse myoblasts (ATCC, Manassas, VA) were cultured in DMEM medium (ThermoFisher Scientific) plus 10% fetal bovine serum, 2% horse serum, 2 mM glutamine, 100 U.I./ml penicillin streptomycin (ThermoFisher Scientific) and 2ug/ml Fungizone (Gemini Bioproducts). Cells were cultured at 37 °C with 5% CO_2_. Cell transfection was performed on Nunc^®^ glass bottom 35 mm dishes (catalog number 150680). Cells were seeded at 2.5 × 10^4^ cells per dish in log-phase growth 24 h before transfection. Lipofectamine LTX (Invitrogen, Carlsbad, CA) was used for transient transfection following manufacturer protocols. Plasmids were expressed by transfecting a total of 1.5 µg of DNA per well. Each transfection was repeated at least three times and each transfection was performed in duplicate. MATR3 and TDP-43 cotransfections were performed in a 1:1 DNA concentration (by wt.). The analysis of MATR3:YFP distribution and morphology was performed by an investigator that was blind to cell genotype.

### Microscopy

Microscopy of fixed cells was performed using a laser-scanning confocal microscope Nikon A1RMP image system with a Nikon A1R scanner, Nikon A1-DUG 4 channel filter based detector unit and Nikon LUNV Laser Launch (6 lines) with a X60 water immersion objective. Cells plated on glass coverslips were washed twice with PBS and fixed with 4% paraformaldehyde post-transfection. Images were processed using Nikon NIS-Elements software v4.5. The excitation/emission wavelengths during acquisition were 488 nm/492–557 nm for YFP

For live imaging, cells were plated on Nunc^®^ dishes (ThermoFisher Scientific). Images and time-lapse videos were captured on a Nikon Eclipse Ti-E Inverted Fluorescent fluorescence microscope image system with an Andor Zyla sCMOS Monochrome camera, Lumencor Spectra X Light Engine and Tokai-Hit Stage Incubation chamber with 40X and 20X objective. Images were collected every 30 s for 24 hours and then processed identically between each condition to adjust brightness and contrast using the Nikon NIS-Elements software v4.5. Each experiment was repeated 3 times, and 5–7 fields were visualized in each experiment.

### Data availability

All RNA-seq data has been submitted to Gene Expression Omnibus (www.ncbi.nlm.nih.gov) accession number GSE110492. The mass spectrometry proteomics data have been deposited to the ProteomeXchang Consortium via the PRIDE[1] partner repository with the data set identifier PXD008896.

## Electronic supplementary material

Supplementary Information

Supplementary Table S1

Supplementary Table S5

Video S1
