# Supplementary material for: Characterization of gene regulation and protein interaction networks for Matrin 3 encoding mutations linked to amyotrophic lateral sclerosis and myopathy
Source: Sci Rep. 2018 Mar 6;8:4049. doi: 10.1038/s41598-018-21371-4 (PMC5840295; doi:10.1038/s41598-018-21371-4)
Supplement: Supplementary file 1 — Supplementary Information [file 41598_2018_21371_MOESM1_ESM.pdf]

Supplementary Information

Characterization of gene regulation and protein interaction networks for Matrin 3 encoding mutations linked to amyotrophic lateral sclerosis and myopathy

M. Carolina Gallego Iradi[1]*, Judy C. Triplett[1]*, James D. Thomas[2]*, Rachel Davila[1], Anthony M. Crown[1], Hilda Brown[1], Jada Lewis[1], Maurice S. Swanson[2], Guilian Xu[1], Edgardo Rodriguez-Lebron[3], David R. Borchelt[1]

[1]Department of Neuroscience, Center for Translational Research in Neurodegenerative Disease, McKnight Brain Institute, University of Florida, Gainesville, FL

[2]Department of Molecular Genetics & Microbiology, Center for Neurogenetics, McKnight Brain Institute, University of Florida, Gainesville, FL

[3]Department of Pharmacology & Therapeutics, Center for Translational Research in Neurodegenerative Disease, McKnight Brain Institute, University of Florida, Gainesville, FL

*These authors contributed equally to this report.

Address correspondence to David R. Borchelt, 1275 Center Dr., Box 100159, Gainesville, FL 32610, email: drb1@ufl.edu

Supplementary Figures S1 – S17

Supplementary Tables S2, S3, S4, S6, and S7.

Supplementary Tables S1 and S5 are provided as separate Excel Files

Supplemental Fig. S1

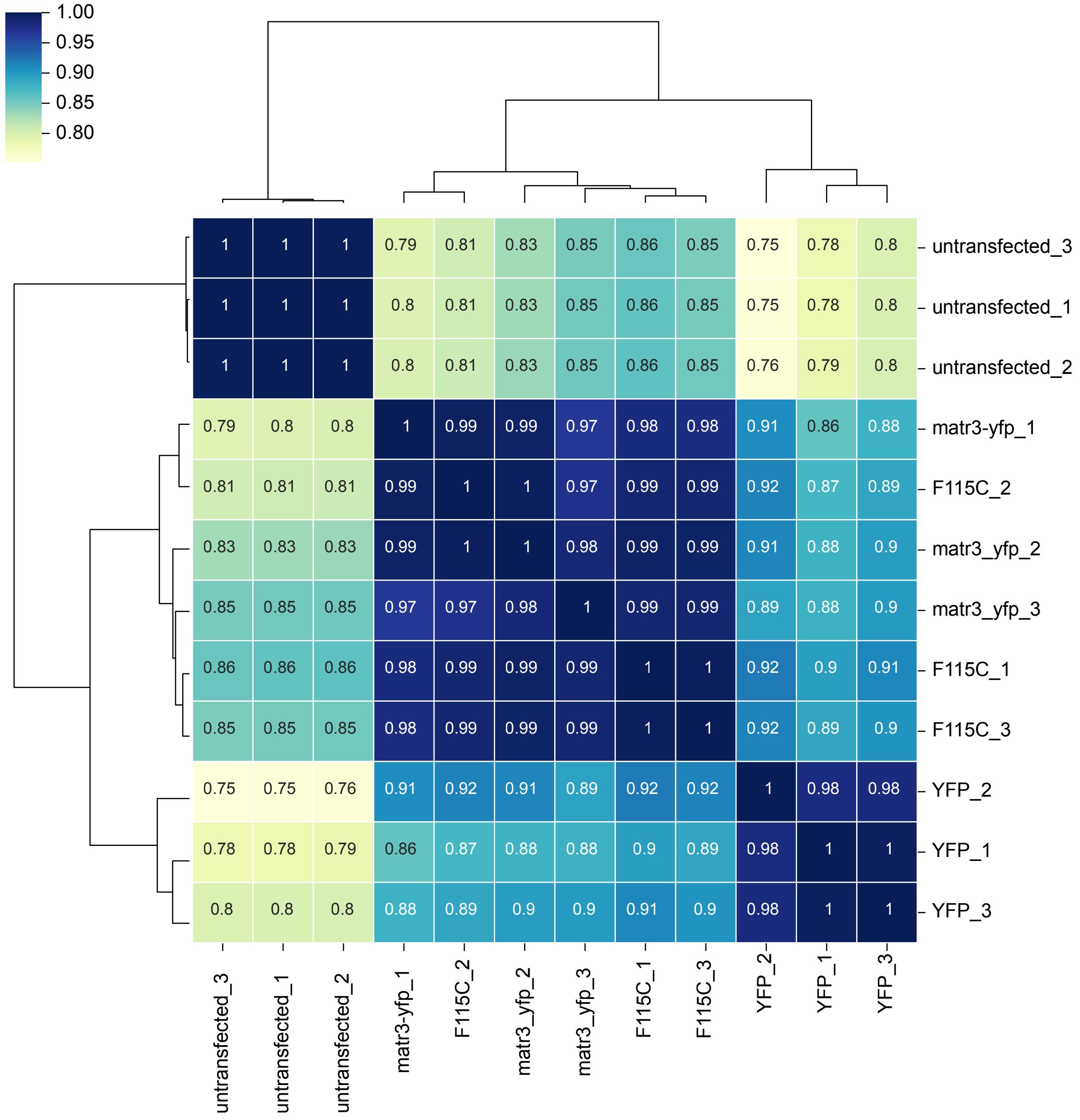

Supplemental Figure S1. Pearson coefficient analysis of RNAseq data.
To assess the variance in RNAseq data from the 3 replicate samples for each condition of transfection, we performed an unsupervised clustering of Pearson correlation values for all pair-wise comparsions.

Supplemental Fig. S2

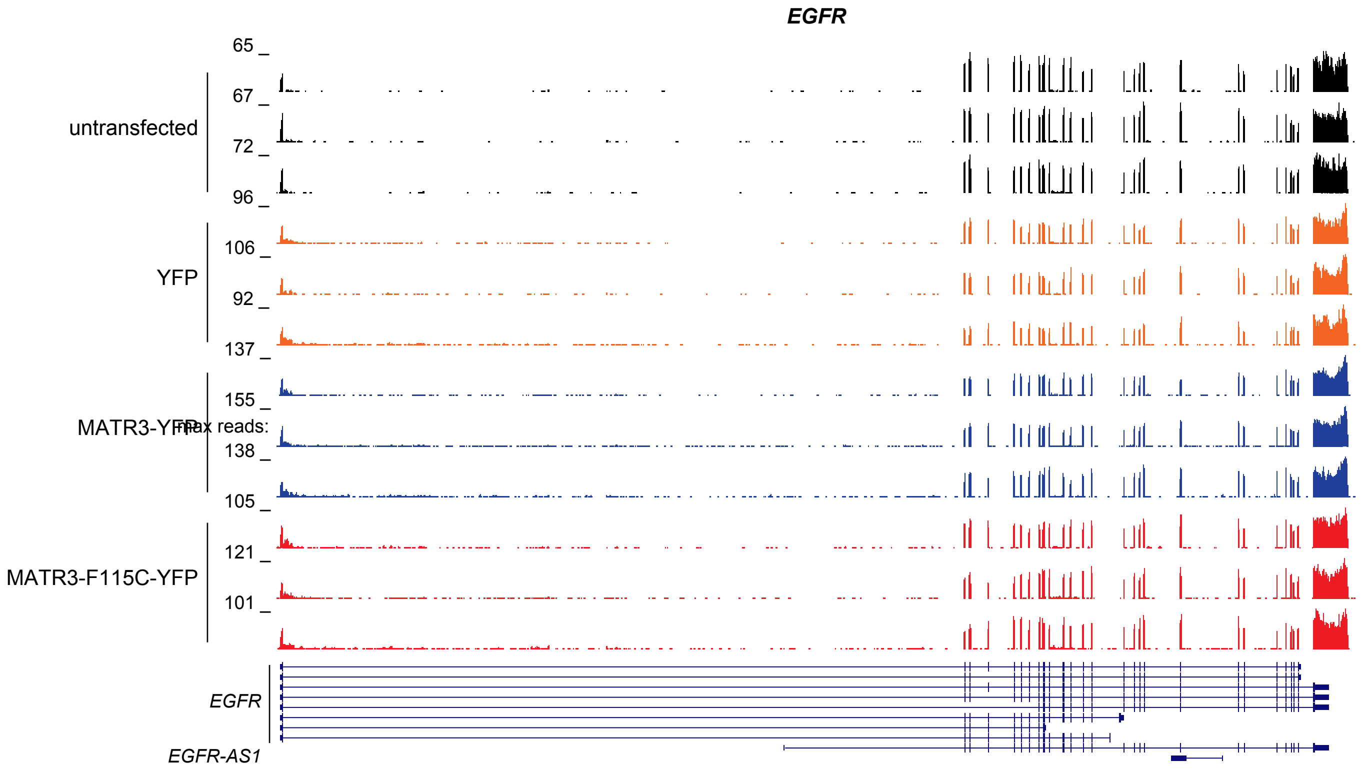

Supplemental Fig. S3

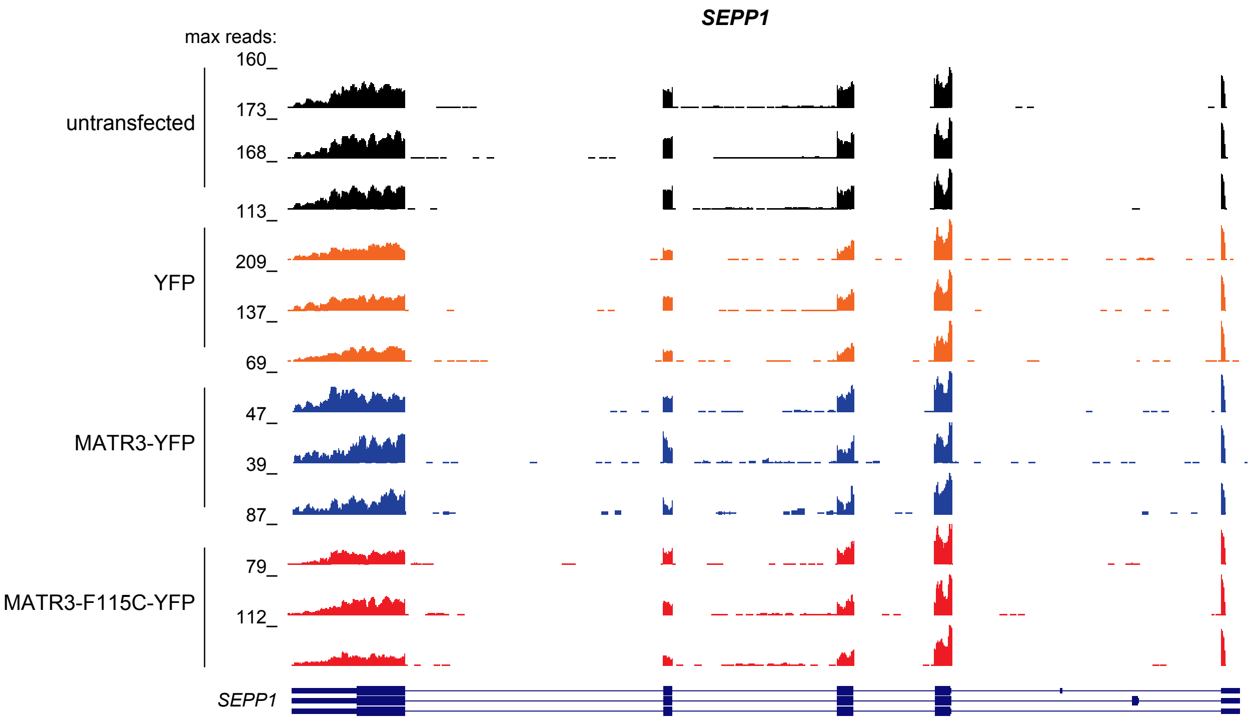

Supplemental Fig. S4

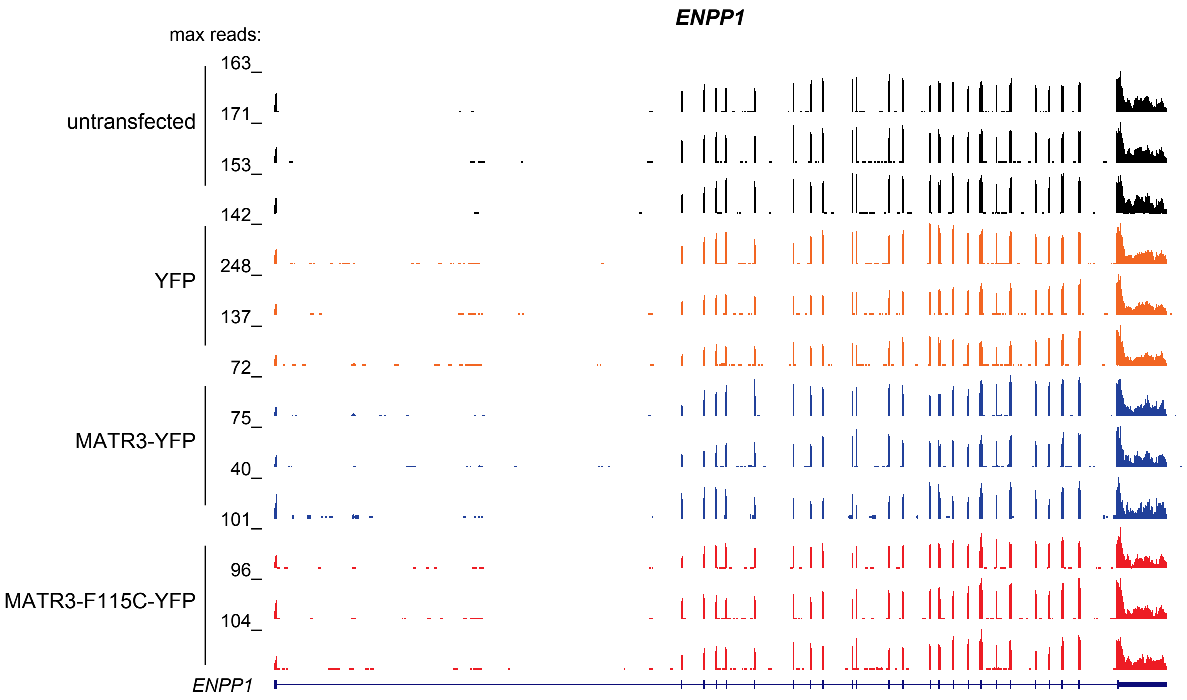

Supplemental Fig. S5

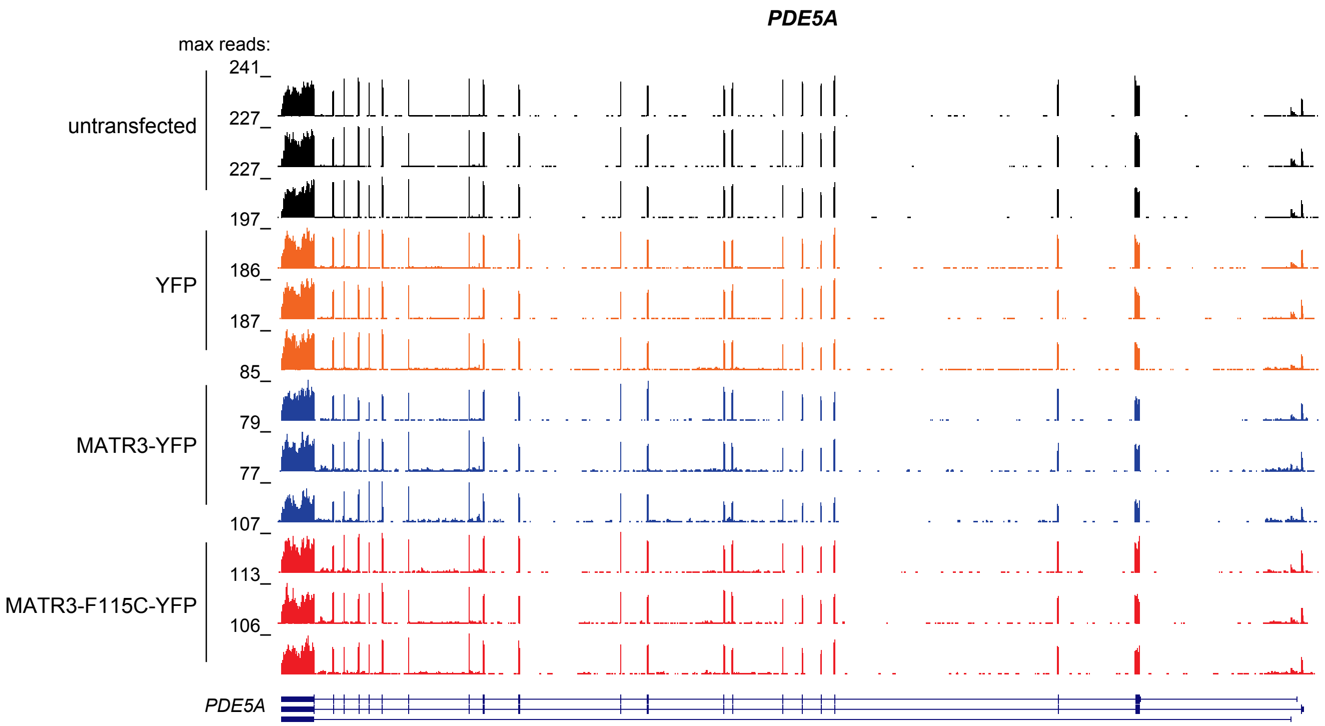

Supplemental Fig. S6

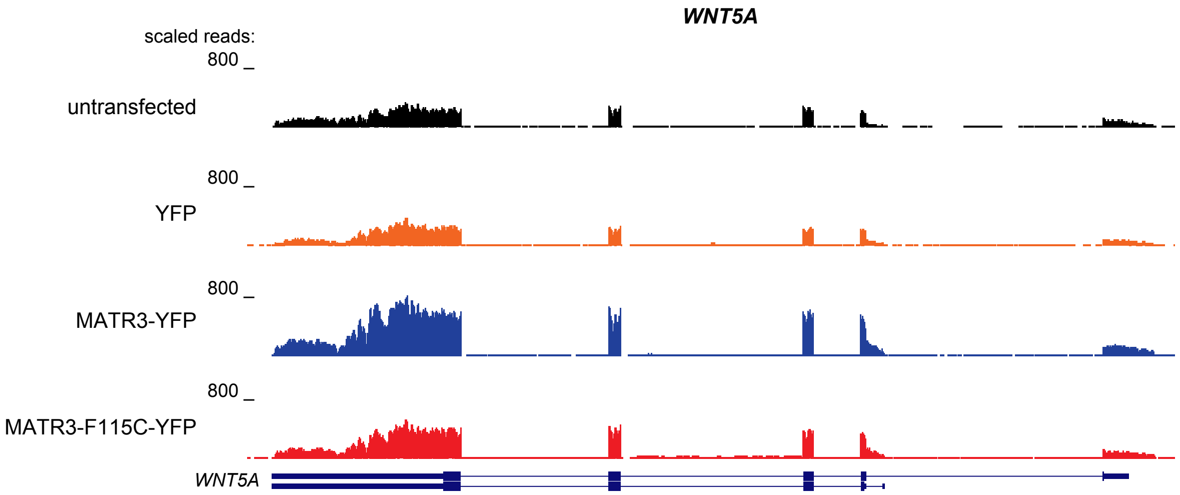

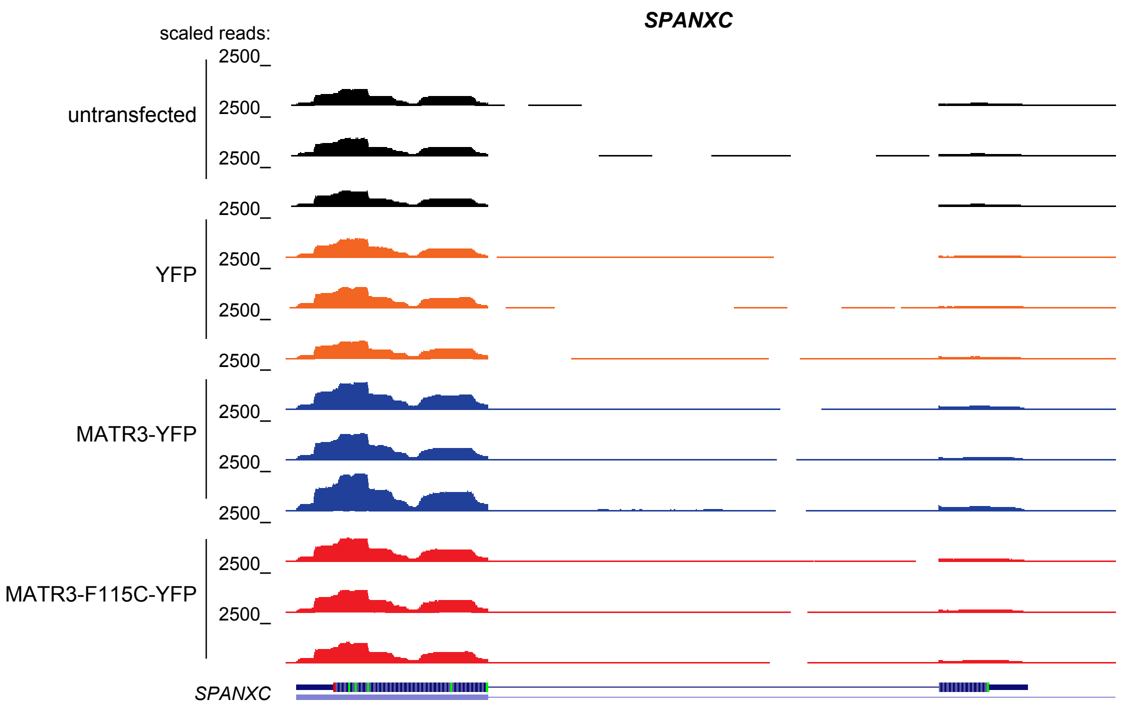
SPANXC
scaled reads:
2500_
2500_
2500_
2500_
2500_
2500_
2500_
2500_
2500_
2500_
2500_
2500_
untransfected
YFP
MATR3-YFP
MATR3-F115C-YFP
SPANXC

Supplemental Fig. S8

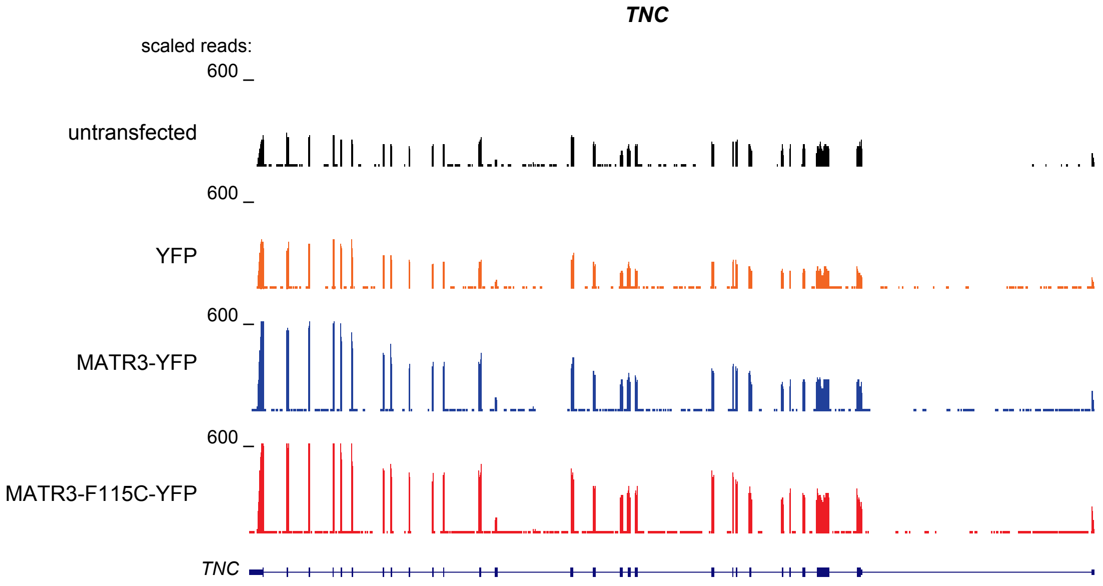

Supplemental Fig. S9

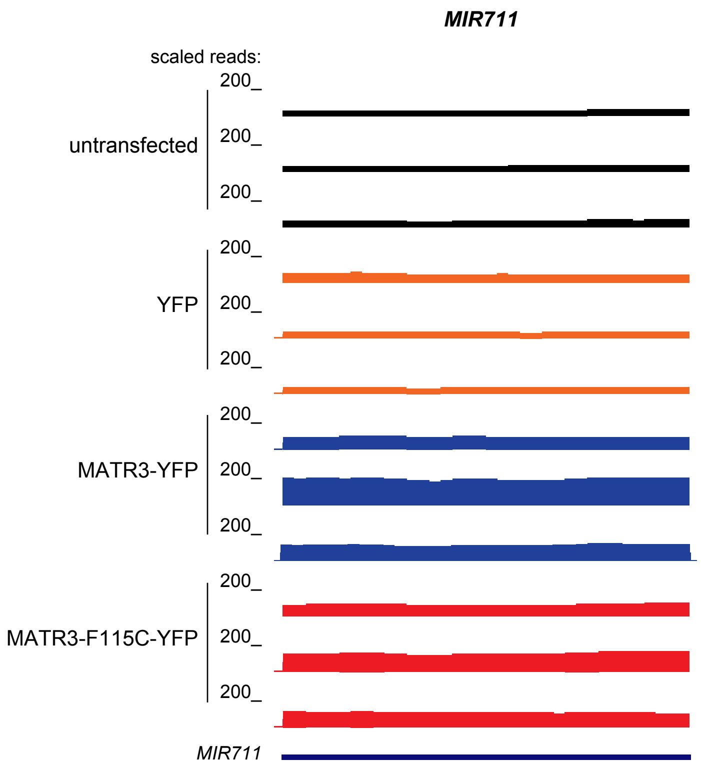

Supplemental Fig. S10

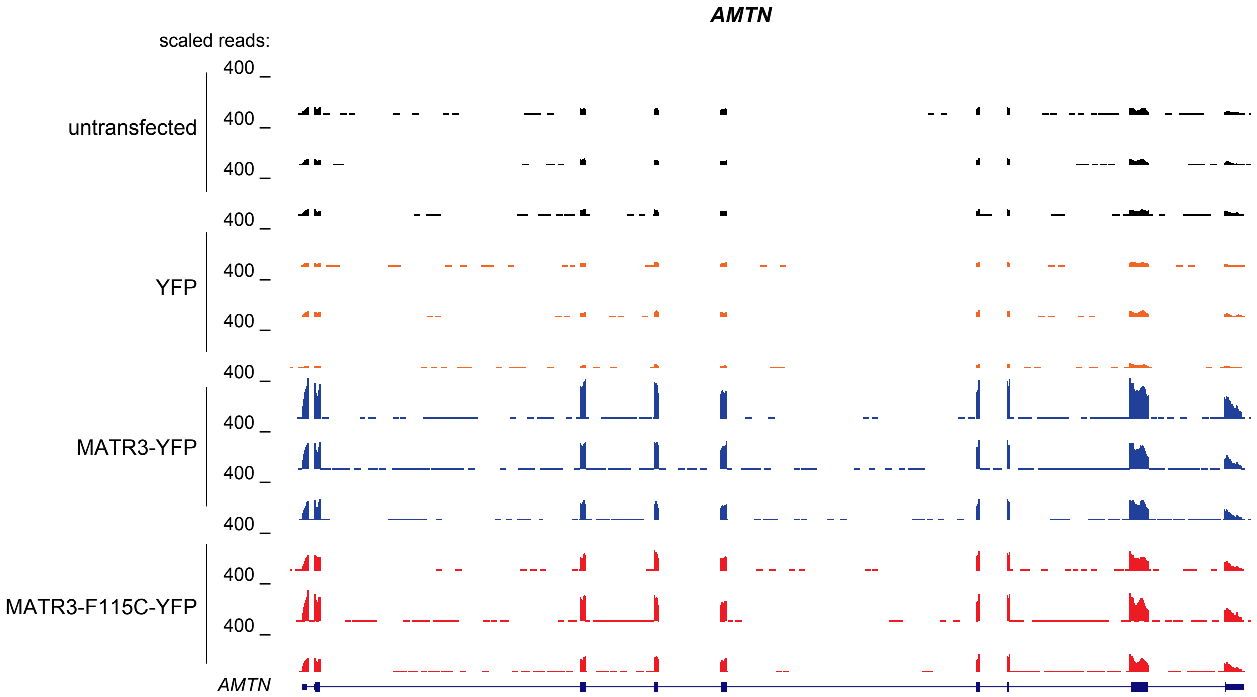

Supplemental Fig. S11

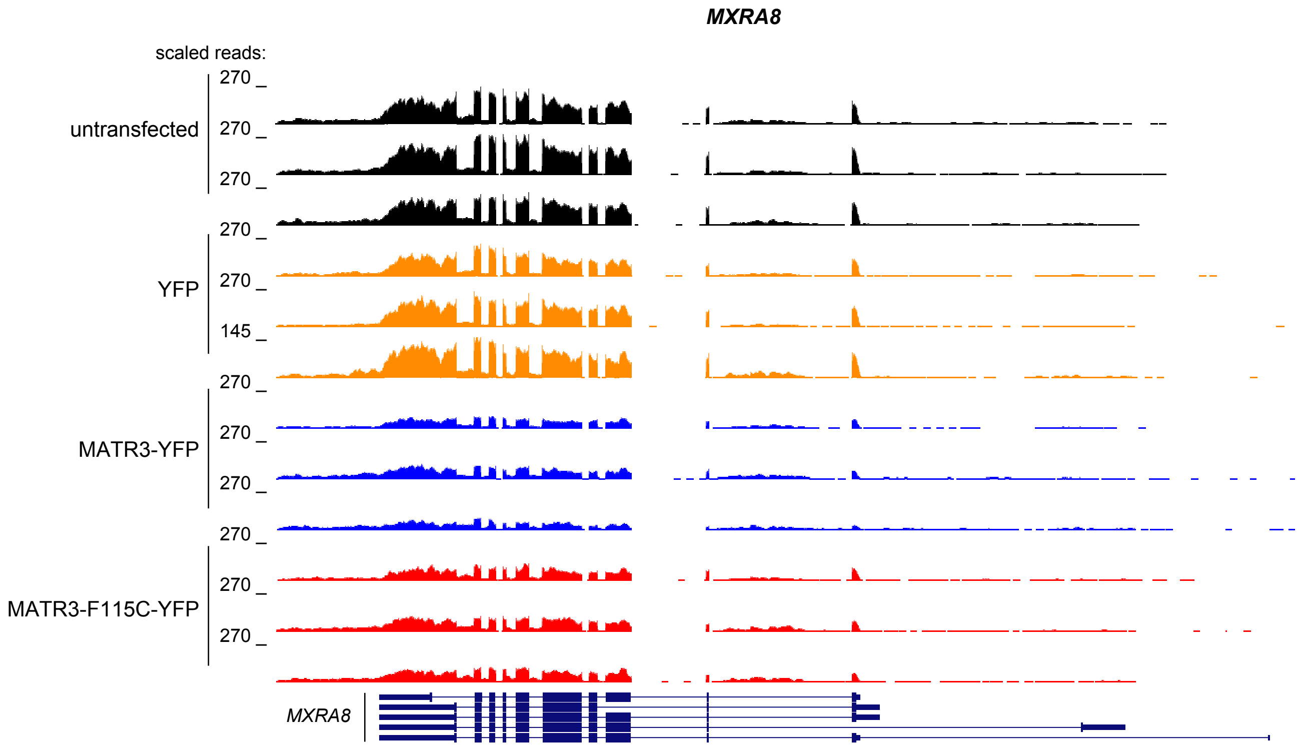

Supplemental Fig. S12

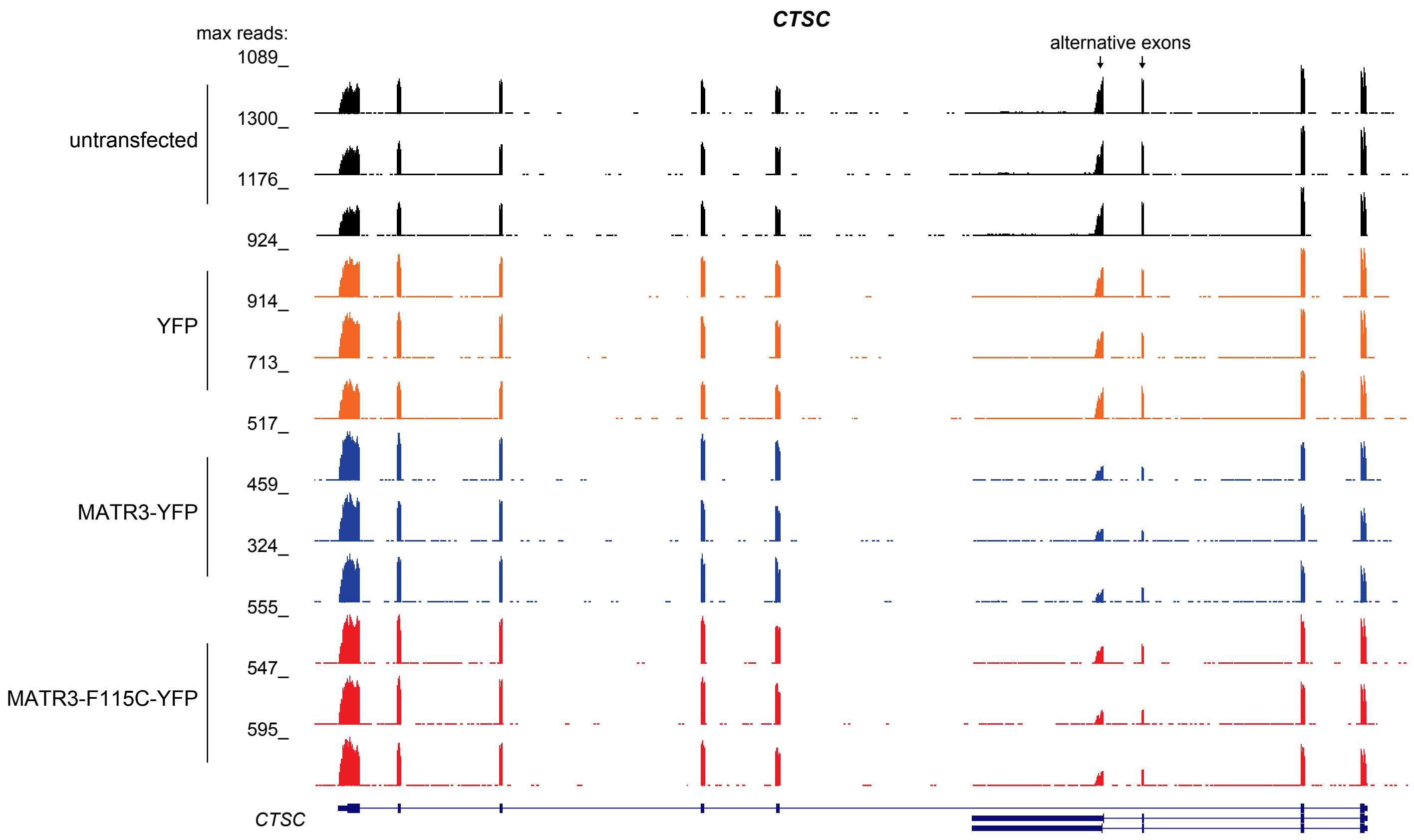

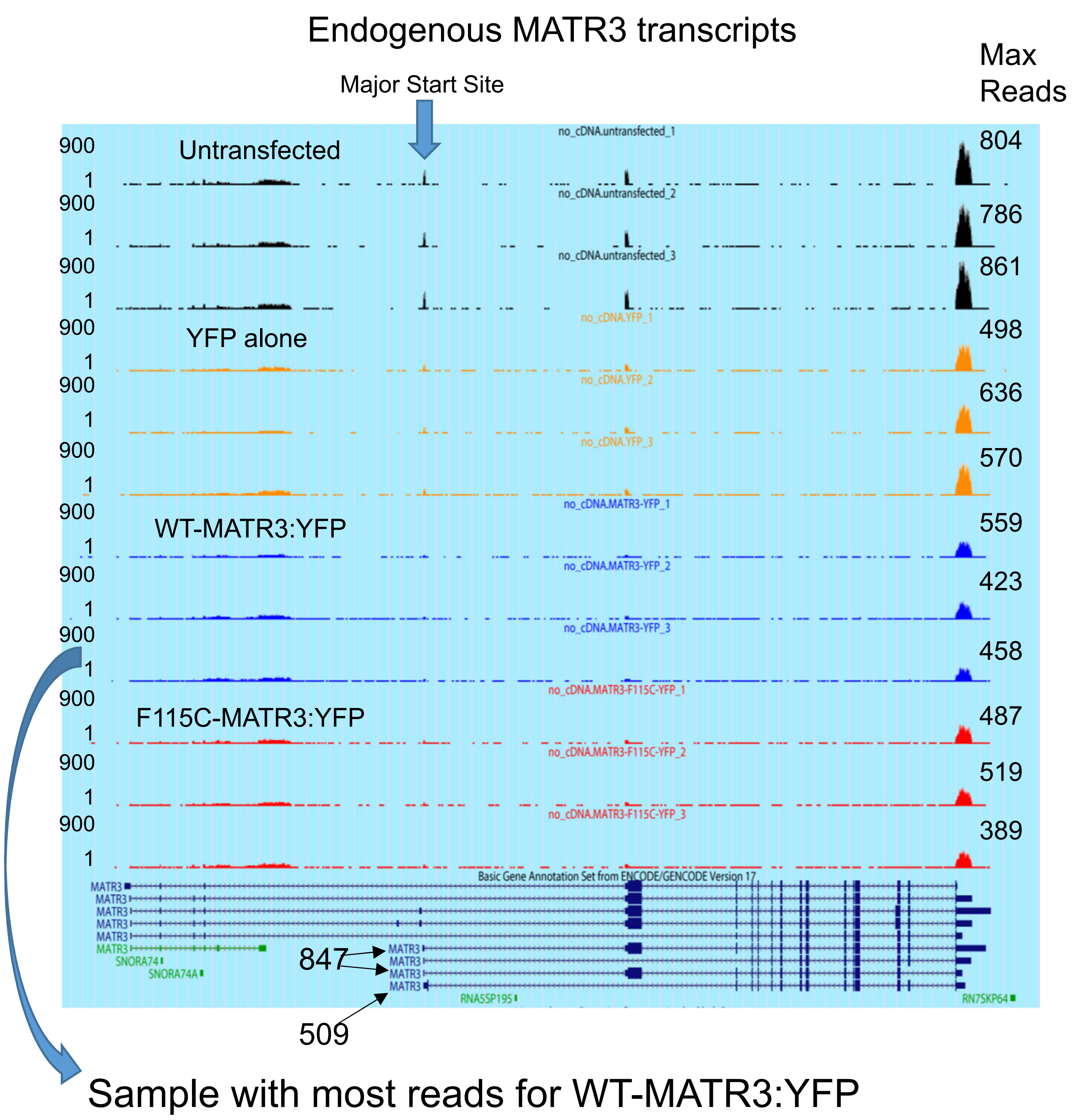

Supplemental Fig S13. Image captured from Genome Browser that shows the read mapping with maximum RPKM for endogenous MATR3 after removal of the reads that would map to the coding exons that overlap with the cDNA for MATR3:YFP.

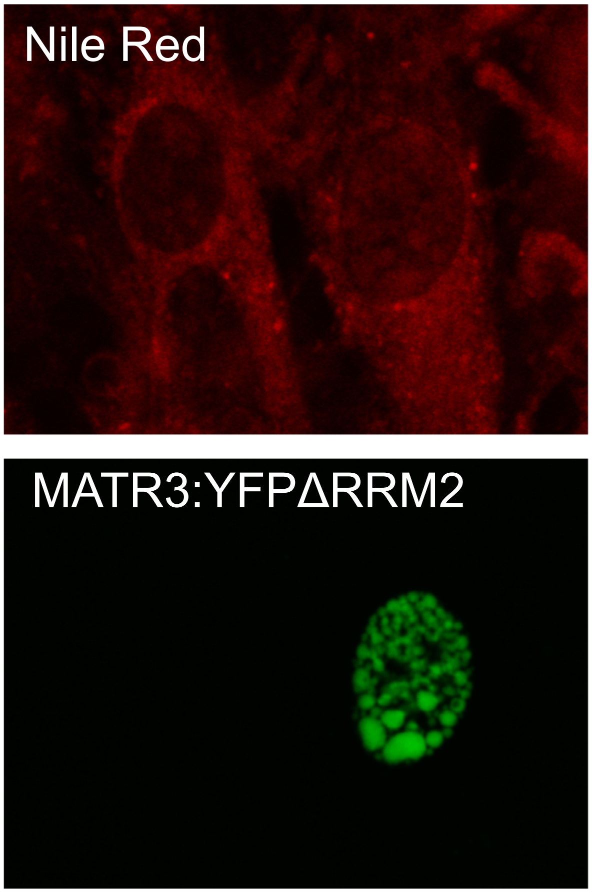

Supplemental Figure S14. Representative example of cells stained with Nile Red. C2C12 cells were transiently transfected with expression plasmids for MATR3:YFPΔRRM2 and then 24 hours later the cells were fixed in 4% paraformaldehyde for 10 minutes before washing in PBS. The cells were stained with Nile Red prepared by diluting a 1 mg/ml stock solution (in Acetone) 1 to 10,000 in PBS before applying to the cells for 5 minutes. After 2 washes in PBS, the cells were imaged on a Nikon Eclipse Ti-E Inverted Fluorescent fluorescence microscope image system as described in Methods at 40X magnification. The image shown is representative of what was observed in 2 independent transfection experiments, visualizing 20-50 cells in each experiment.

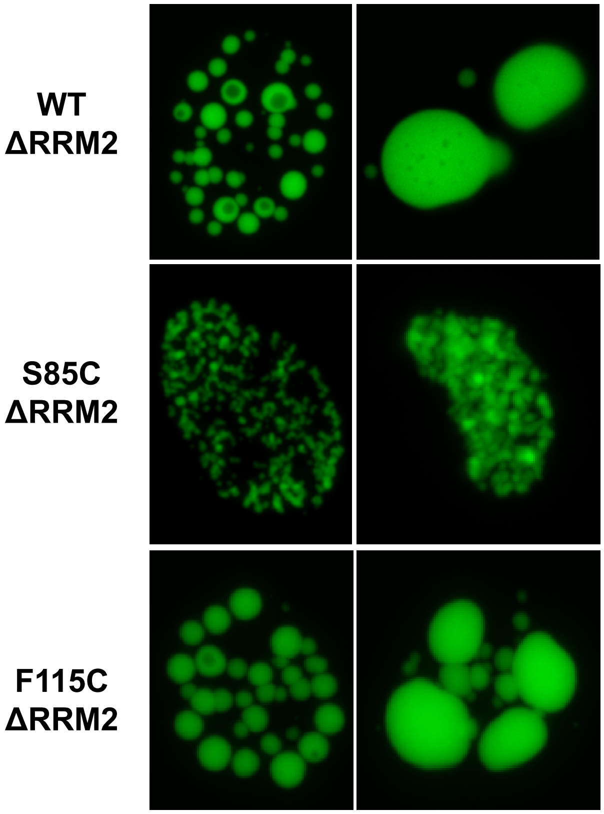

Supplemental Figure S15. Disease mutations produce different effects on the formation of spheres by MATR3:YFPΔRRM2. Representative images of C2C12 cells expressing WT, S85C, and F115C MATR3:YFPΔRRM2. The number of cells analyzed in 3 separate transfection experiments is provided in Table 2. In a single Z-plane, some spheres appear as ring-like structures, or contain a relatively large vacuole. Larger spheres contain small vacuoles, or bubbles. The original magnification of image capture was 60x, with some digital enlargement.

Supplemental Fig. S16

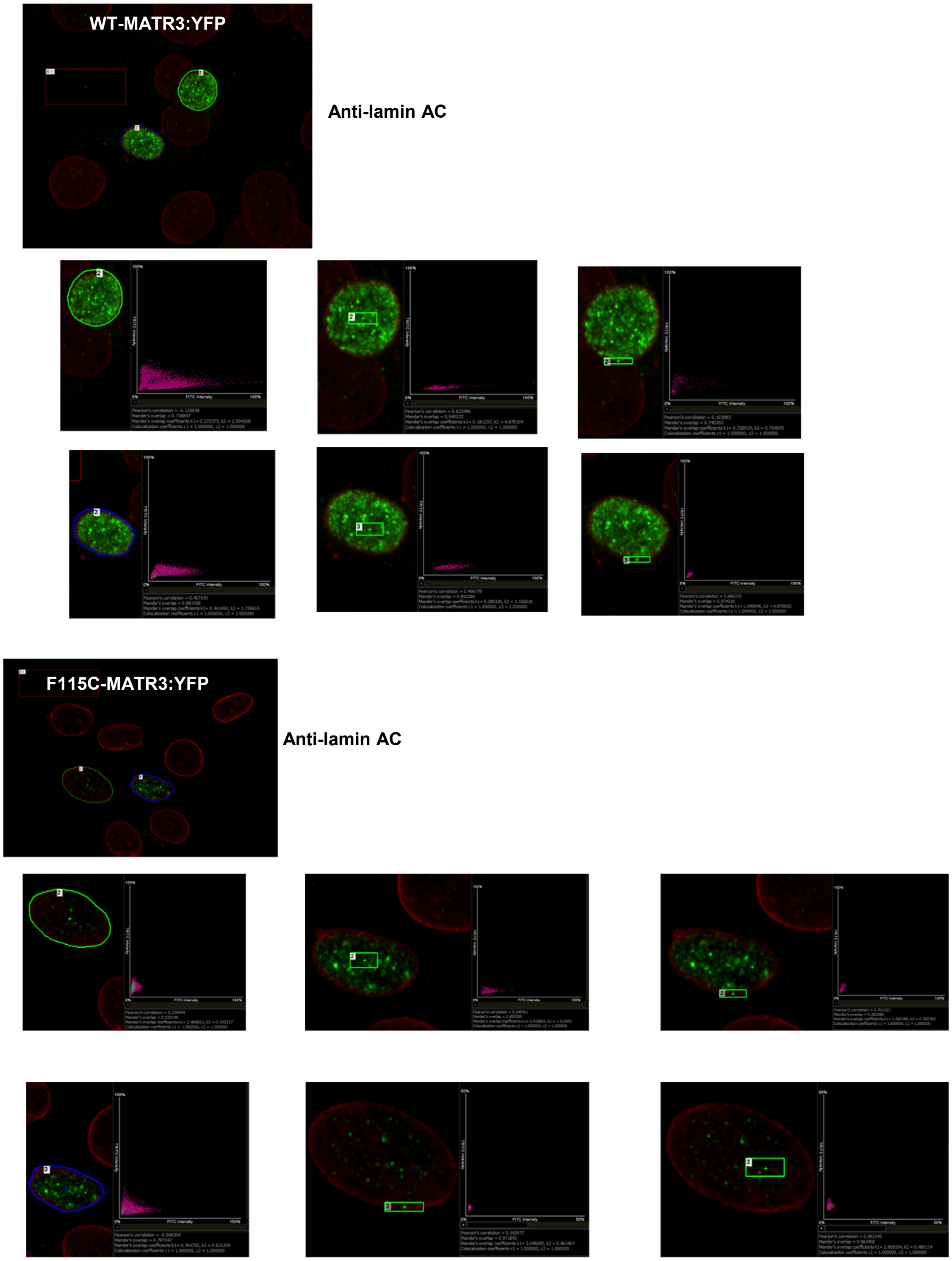

Supplemental Figure S16. Analysis of MATR3:YFP co-localization with lamin A/C. Representative images of C2C12 cells expressing WT and F115C MATR3:YFP co-stained with antibodies to lamin AC (red). The degree of fluorescence co-localization in a single Z-plane was assess using Nikon elements software. The original magnification of image capture was 60x, with some digital enlargement.

Supplemental Fig. S17

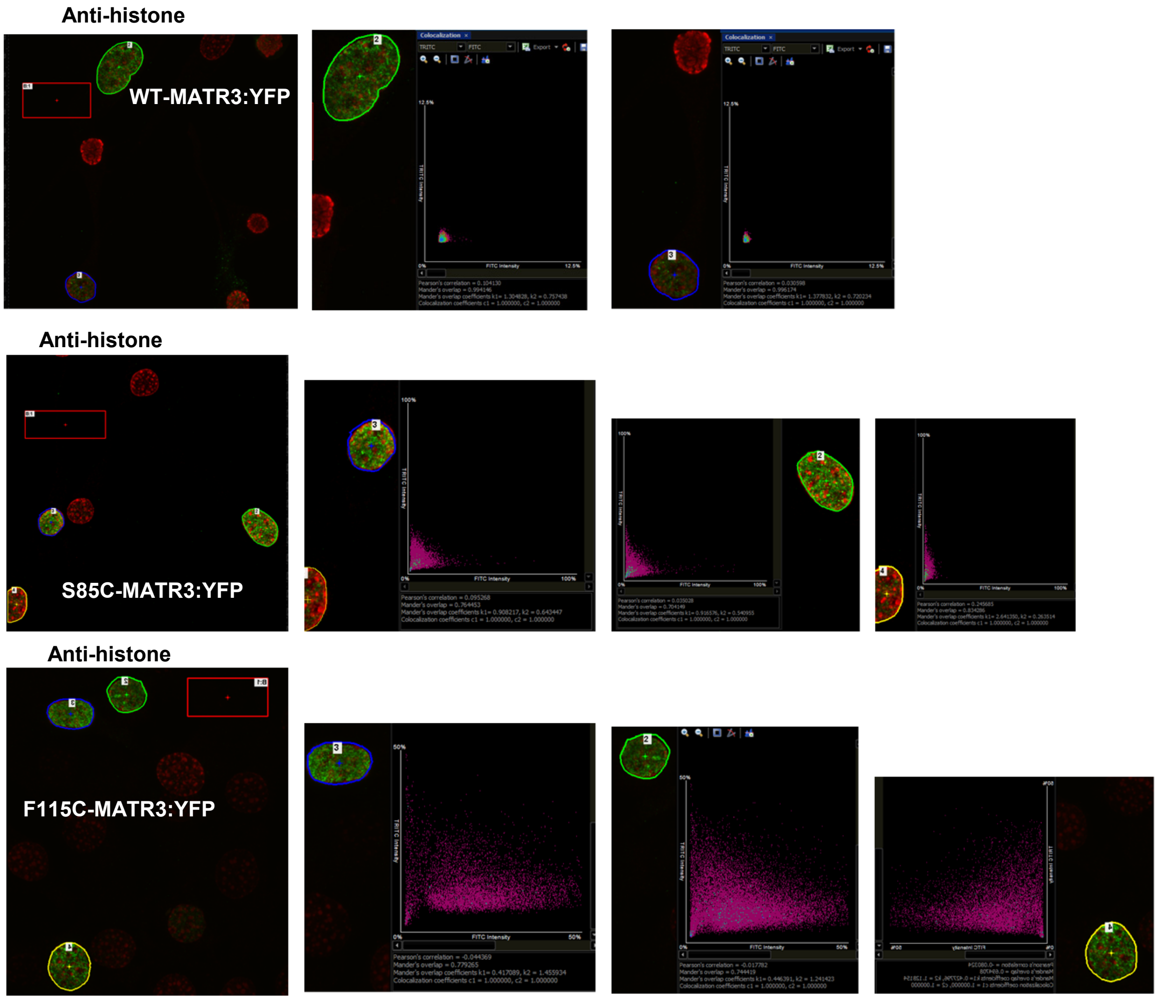

Supplemental Figure S17. Analysis of MATR3:YFP co-localization with histone. Representative images of C2C12 cells expressing WT and F115C MATR3:YFP co-stained with antibodies to histone (red). The degree of fluorescence co-localization in a single Z-plane was assess using Nikon elements software. The original magnification of image capture was 60x, with some digital enlargement.

Supplemental Table S2. Genes influenced by over-expression of MATR3:YFP		
Gene Name	Log 2 fold change WT MATR3:YFP vs YFP	Fold change F115C MATR3:YFP vs YFP
Genes induced by YFP expression with further induction by WT or F115C MATR3 expression (log FC relative to untransfected)		
ORM1	3.50 (6.49)	2.48
SERPINB2	3.23 (4.69)	1.70
FDCSP	2.87 (7.12)	2.22
CSF2	2.59 (11.32)	2.04
SPRR2D	2.32 (6.30)	2.05
EID3	2.30 (4.09)	1.90
OLR1	2.08 (3.93)	1.68
SAA1	1.96 (9.40)	1.68
TNFAIP6	1.95 (8.59)	1.42
TNFAIP2	1.91 (5.13)	1.81
C15orf48	1.87 (8.73)	1.16
SAA2	1.68 (10.51)	1.56
SERPINA1	1.63 (2.79)	1.09
CXCL11	1.63 (12.66)	1.20
CCL2	1.62 (7.03)	1.40
CX3CL1	1.54 (4.63)	1.48
SERPINE2	1.54 (2.43)	1.15
COL7A1	1.51 (2.03)	1.36
SLC2A6	1.45 (3.21)	1.39
IL8	1.42 (8.85)	1.13
IL32	1.41 (7.27)	1.18
IL6	1.28 (10.99)	1.04
TNFRSF9	1.26 (6.04)	1.29
DNER	1.25 (2.02)	0.90
INHBA	1.24 (6.43)	0.84
KLHDC7B	1.23 (9.41)	1.23
CCL20	1.21 (10.64)	0.98
SPANXE	1.16 (2.17)	0.65
ELF3	1.16 (1.88)	1.14
CXCL3	1.11 (8.99)	1.02
EDN1	1.07 (4.02)	0.50
C3	1.07 (4.04)	1.18
MT1X	1.06 (2.26)	0.60
CCL3	1.04 (12.08)	N.S.
CXCL10	1.03 (11.68)	0.99
BIRC3	1.02 (3.65)	0.91
CD82	0.99 (3.65)	0.64
IL7R	0.98 (1.92)	0.74
RAB27B*	-0.97 (2.43)	-0.83
RARRES3*	-0.98 (1.90)	-0.92
HIST1H2BC*	-0.99 (1.00)	N.S.
CTGF*	-1.01 (2.87)	-0.69
CA9*	-1.02 (4.65)	-0.82
TDO2*	-1.03 (2.78)	N.S.
IL1R1*	-1.06 (0.81)	-0.68
VWA5A*	-1.08 (2.15)	-0.64
LOX*	-1.08 (1.56)	-0.67
LGALS9*	-1.09 (4.91)	-0.97
TIMP3*	-1.18 (0.45)	-0.82
A2M*	-1.18 (3.11)	-0.88
TGFA*	-1.18 (0.88)	-0.93
AGT*	-1.20 (2.42)	-0.96

TGM2*	-1.22 (1.38)	-1.06
IFNB1*	-1.30 (9.44)	-0.91
FOS*	-1.34 (4.36)	-0.72
IL18BP*	-1.40 (2.18)	-1.30
MGP*	-1.47 (0.73)	-1.33
PLAT*	-1.73(2.5)	-1.33
CD74*	-2.33(4.14)	-2.25
Genes suppressed by YFP expression		
HBE1	1.13 (0.53)	0.98
Genes suppressed by WT or F115C MATR3 Overexpression		
Genes induced by YFP expression		
TRIB2	-1.20 (-0.62)	-0.84
Genes suppressed by YFP		
UNC13D	-0.96 (-0.59)	0.79
CRISPLD1	-0.96 (-3.38)	-0.45
ARHGAP28	-0.99 (-2.70)	N.S.
CAT	-1.00 (-1.55)	-0.72
ENPP2	-1.03 (-2.16)	N.S.
FLJ22447	-1.07 (-1.54)	-0.61
COLEC12	-1.08 (-2.58)	-0.56
EGLN3	-1.14 (-1.97)	-0.73
RASL11A	-1.23 (-3.96)	N.S.
PDGFRA	-1.35 (-1.73)	-0.92
VAV3	-1.39 (-2.26)	-1.93
PI15	-1.47 (-4.42)	-0.43
AKAP6	-1.57 (-2.84)	-0.89
ITGA8	-1.59 (-2.72)	-0.90
PBX1	-1.72 (-2.90)	-1.12
CBLN2	-2.20 (-4.88)	-1.40
*Suppressed by WT and F115C MATR3 relative to YFP expressing cells but induced relative to untransfected cells.		

**Supplemental Table S3. Non-coding RNA detected in RNA-seq dataset**		
**Non coding RNAs**	**Untransfected vs YFP**	**Untransfected vs WT MATR3:YFP**
tRNAs	1	1
mt-tRNAs	21	21
rRNAs	1	1
miRNAs	64	64
Piwi RNAs	0	0
SCARNA	23	23
Small nucleolar RNAs (SNORD)	189	191
Small nucleolar RNAs (SNORA)	76	76
Long non coding RNAs	105	105
Linc RNAs	0	0
ERV	4	4
DNA transposon derived genes transposable	21	21
Gag like LTR retrotransposon	55	55
Competing endogenous long non coding RNAs (CERNA)	8	8
Transcribed pseudogenes	10	10
Pseudoautosomal region 1	20	20
Non coding RNA Ro associated Y	0	2
Non coding RNA 7sl cytoplasmic (RN7SL)	0	0
Small nuclear RNAs non coding	20	20
Cytoplasmic transfer RNAs (TR)	103	103
Mitochondrially encoded tRNAs (MT-T)	1	1
Nuclear encoded mitochondrial tRNAs	0	0
Vault RNAs (VTRNA)	6	6

Supplemental Table S4. Proteins binding WT MATR3 and MATR3 deletion mutants.

Protein (location/pathway)	MW kDa	RRM	PrLD	Avg # spectra control	Avg # spectra WT	Fold Change vs Control	p Value	Ratio ΔRRM1 vs WT	Ratio ΔRRM2 vs WT	Ratio ΔZnf1 vs WT	Ratio ΔZnf2 vs WT	SAINT Score	SAINT Fold Change
MATR3	100	2	LC	5.4	375	69	0.02316	-	-	-	-	-	-
**HNRNPM (N,No,I)**	40	2	LC	4	157	40	0.03967	1.22	0.62	1.30	1.76	1	39.4
SFRS14 (N,S)	120	other†	LC	1*	26	26	0.00018	0.32	0.38	0.32	0.76	1	262.5
**PTBP3(N,S,SG)**	57	4	-	1*	25	25	0.00518	0.69	0.51	0.98	0.78	1	61.3
M0QYT0 Unc	36	1	-	1*	24	24	0.00015	0.59	0.74	0.45	0.97	1	29.7
**ADAR (N,P,SG)**	138	other†	-	1*	23	23	0.01720	0.13	0.38	0.05	0.40	1	55.6
PRPF8 (N,No,S)	274	other†	-	1	20	20	0.00693	0.28	2.60	0.15	1.15	1	20.0
A0A024R8A7 Unc	62	other†	Y	2	39	19	0.0000003	0.55	0.52	0.37	0.77	1	19.4
**PTBP1 (N,S)**	59	4	-	2.8	52	19	0.01502	0.90	2.32	1.23	1.45	1	18.7
**ZNF326 (N,S)**	66	-	-	1*	18	18	0.000011	0.38	0.11	0.49	0.38	1	182.5
PABPC1 (N,P,SG)	71	4	Y	1.4	25	18	0.00599	0.58	0.80	0.62	1.32	1	17.9
**ILF3 (N,No,I)**	96	other†	Y	1.8	32	18	0.000001	0.61	1.03	0.53	1.16	1	17.8
PABPC4 (N,P)	70	4	Y	1*	18	18	0.00002	0.48	0.51	0.34	0.90	1	44.4
SNRNP200 (N,S)	245	other†	LC	1*	17	17	0.02466	0.24	3.15	0.07	1.03	1	20.6
MYBBP1A (N,No,T)	149	-	LC	1*	16	16	0.00057	0.37	3.45	0.52	1.48	1	81.3
B2R959 *HNRNPL sim*	60	3	LC	2	31	16	0.00026	0.61	0.77	0.68	1.03	1	15.5
**HNRNPA2B1 (N,P,SG)**	37	2	Y	5.8	88	15	0.00826	0.62	1.79	1.09	0.94	1	15.1
DHX30 (M,R,SG)	134	other†	-	1*	15	15	0.01069	0.13	0.77	0.20	0.40	1	150.0
IGF2BP3 (N,P,SG)	64	2	-	1*	15	15	0.00005	0.28	1.45	0.17	0.41	1	72.5
BCLAF1 (N,P)	106	-	LC	1*	14	14	0.000001	1.05	1.93	0.87	1.82	1	34.4
**HNRNPA3 (N,S,SG)**	40	2	Y	2.4	32	13	0.00003	0.63	1.41	0.86	1.69	1	13.3
SYNCRIP (N,No,S,SG)	70	3	LC	1.2	16	13	0.00022	0.22	1.31	0.60	0.63	1	13.3
A0A024RC46 Unc	34	2	Y	3.6	48	13	0.00114	0.48	2.58	0.95	1.47	1	13.3
**HNRNPA1 (N,S,SG)**	34	2	Y	4.4	57	13	0.00191	0.57	2.34	1.06	1.57	1	13.0
MOV10 (SG)	114	other†	-	1*	13	13	0.01766	0.08	0.27	0.14	0.15	1	130.0
ATAD3B (M)	73	-	LC	1	13	13	0.01226	0.76	0.88	1.49	2.08	1	15.6
**HNRNPUL1 (N,S,SG)**	90	other†	Y	1*	13	13	0.00465	0.08	0.84	0.10	0.16	1	62.5
THRAP3 (N,S)	109	other†	Y	1*	12	12	0.000003	0.92	0.92	0.45	1.08	1	120.0
DDX1 (N,No,P,T)	82	other†	-	1*	12	12	0.01004	0.13	0.04	0.05	0.68	1	29.4
**HNRNPR (N,S,P)**	71	3	Y	1.6	18	11	0.00006	0.27	1.81	0.56	1.10	1	11.4
RPL13A (R,I)	24	-	LC	1*	11	11	0.00047	0.91	2.41	1.75	2.82	1	13.8
RPL15 (N,No,R)	24	-	LC	1*	11	11	0.00014	0.57	2.48	0.74	2.00	1	52.5
RPL6 (N,No,R)	33	-	LC	2	21	10	0.00043	0.48	2.39	1.07	2.12	1	10.4
GTPBP4 (N,No,R)	74	-	LC	1*	10	10	0.00054	0.20	1.61	0.06	1.07	1	51.3
**HNRNPUL2 (N)**	85	-	Y	1*	10	10	0.00052	0.10	0.59	0.12	0.29	1	102.5
RSL1D1 (N,No,P,SG)	55	-	LC	1*	10	10	0.00263	0.15	4.40	0.42	2.20	1	25.0
SMC1A (N,No,Ch)	143	-	LC	1	10	10	0.01218	0.30	3.15	0.24	0.50	0.99	10.0
B0AZQ4 (N,Ch)	142	-	LC	1*	10	10	0.02789	0.15	3.03	0.06	0.21	1	48.8
MYEF2 (N,T)	64	3	LC	1*	10	10	0.00366	0.10	0.36	0.31	0.21	1	97.5
SMCHD1 (N,No,Ch)	226	-	-	1*	10	10	0.03239	0.15	3.23	0.25	1.85	1	24.4
IGF2BP1 (N,P,SG)	63	2	-	3.5	33	10	0.000003	0.55	1.06	0.49	0.79	1	9.7
EPPK1 (SG)	556	-	-	2.2	21	10	0.00753	0.56	1.22	0.51	2.26	0.99	8.9
**KHDRBS1 (N,No,S,P,SG)**	48	other†	LC	1.6	15	9.5	0.00016	0.56	0.98	0.71	1.38	1	9.5
DHX15 (N,No,S)	91	other†	LC	2	19	9.5	0.000008	0.24	1.13	0.32	1.11	1	9.5
B3KY60 (N,T) ***CDC5L sim***	92	-	LC	1*	9.5	9.5	0.00718	0.42	1.26	0.06	0.21	1	11.9
PRPF6 (N,S)	107	-	LC	1*	9.5	9.5	0.00914	0.11	0.84	0.06	0.63	1	47.5
**ILF2 (N,No,I)**	39	-	LC	1.8	17	9	0.00049	0.36	1.46	0.64	0.96	1	9.3

LRPPRC (N,M,P)	158	other†	-	1*	9	9	0.01603	0.16	1.78	0.06	0.43	1	11.6
A8K4T9 (N,S,P) ***RALY* sim**	30	1	Y	1*	9	9	0.00004	0.44	1.22	1.20	1.56	1	9.0
EIF3S8	105	-	LC	1*	9	9	0.02137	0.06	0.57	0.07	0.23	1	87.5
B2RCM2 *LARS sim*	134	-	LC	1*	9	9	0.00893	0.06	0.46	0.41	0.46	1	87.5
RPS9 (N,No,R)	23	other†	LC	2.2	19	8.5	0.00378	0.72	0.96	1.54	1.97	1	8.5
**HNRNPK (N,T,S,P,SG)**	49	other†	LC	5.2	44	8.5	0.01622	0.75	1.72	1.00	1.08	1	8.5
Q59GX6 helicase	75	-	Y	5.6	48	8.5	0.00266	0.72	1.49	0.73	1.45	1	8.5
RPL7A (R)	30	-	LC	3	25	8.5	0.0004	0.77	1.37	1.52	2.30	1	8.4
RPL28 (R)	16	-	LC	1.2	10	8	0.0349	0.90	1.30	1.44	2.10	0.96	8.3
**RTCB (N,P)**	55	-	-	1.6	13	8	0.00007	0.34	1.17	0.95	1.21	1	8.3
**HNRPH3 (N,S)**	37	2	Y	1	8	8	0.00014	0.36	2.18	0.44	0.85	1	8.3
SF3B2 (N,S)	100	other†	LC	1	8	8	0.01897	0.12	0.18	0.15	0.24	0.96	8.3
**RPL7 (R)**	30	-	LC	3.2	26	8	0.00005	0.54	1.62	1.15	2.19	1	8.1
**SRSF9 (N,S)**	26	2	-	1*	8	8	0.00007	0.58	1.74	1.86	2.06	1	19.4
Q59G75 (*Unc*)	146	-	-	1.2	9	8	0.03206	0.22	1.03	0.32	0.86	0.98	7.7
RPL18 (N,No,R)	22	-	LC	1.2	9	8	0.00574	0.76	3.24	1.75	3.89	0.99	7.7
RPL4 (N,No,R)	48	-	LC	4.4	34	8	0.00109	0.73	1.93	0.96	2.40	1	7.7
ATAD3A (M,SG)	66	-	LC	2.2	17	8	0.00742	0.69	1.01	1.22	1.91	0.99	7.6
A8KAP3 *EFTUD2 sim*	109	-	LC	1.8	13	7	0.02806	0.15	1.89	0.14	0.75	0.97	7.4
Q2VIN3 *RBM1 sim*	41	1	LC	3.2	23	7	0.00213	1.05	0.92	1.21	1.63	1	7.1
**SAFB (N,T)**	103	1	Y	1*	7	7	0.00064	0.71	0.21	0.26	0.43	1	70.0
**YBX1 (N,S,SG)**	36	other†	LC	1*	7	7	0.00128	0.36	1.00	0.43	0.57	1	35.0
DDX21 (N,No,P,SG)	87	other†	LC	3.6	25	7	0.00008	0.34	2.44	0.74	2.64	1	6.9
NOP2 (N,No,R)	89	-	LC	1.6	11	7	0.01063	0.32	1.55	0.44	1.73	0.99	6.9
RPS2 (R)	31	other†	LC	2.6	18	7	0.00215	0.70	1.52	1.22	2.82	1	6.8
ABCF1	96	-	LC	1*	7	7	0.02019	0.37	2.22	0.27	0.89	0.98	8.4
EIF4G1 (SG)	172	-	Y	1*	7	7	0.02074	0.52	1.33	0.27	0.30	1	33.8
RBM4 (N,No,S,P)	40	2	-	1*	7	7	0.00595	0.07	0.59	0.27	0.30	1	67.5
SRSF7 (N,S)	26	1	LC	1*	7	7	0.00995	1.26	1.70	1.16	2.22	0.99	11.3
A8K622 *STAU sim*	63	other†	-	1*	7	7	0.01229	0.15	0.96	0.27	0.44	1	33.8
**DDX17 (N,No,S,P)**	80	other†	Y	8.4	56	9	0.00184	0.80	1.20	0.96	1.72	1	6.7
**HNRNPA0 (N,No,P,I)**	31	2	Y	2	13	7	0.00308	0.42	2.11	1.09	1.89	0.99	6.6
ELAVL1 (N,No,P,SG)	39	3	-	3	20	6.5	0.00593	0.79	0.95	1.20	1.54	0.99	6.5
A8K588 (N,S) *SFRS6 sim*	39	2	LC	1*	6.5	6.5	0.00324	0.85	1.46	1.29	2.15	1	16.3
**HNRNPC (N,S,P)**	32	1	LC	8	51	6	0.00631	0.79	1.32	0.93	1.73	0.99	6.3
RPS3A (N,No,R)	30	-	-	5	32	6	0.00373	0.73	0.83	1.09	1.97	0.99	6.3
RPS13 (N,No,R)	17	-	-	2.6	16	6	0.00108	0.58	1.05	1.51	2.28	1	6.3
SMC2L1 (N,No,C)	136	-	LC	1.4	9	6	0.02893	0.17	2.97	0.27	0.69	0.96	6.3
SFRS3 (N,S,P,SG)	19	1	LC	1.2	8	6	0.00368	0.93	3.07	1.52	2.27	0.99	6.3
B4DMY3 *HNRNPAB sim*	35	2	Y	1*	6	6	0.00007	0.48	2.80	0.77	0.80	1	62.5
TSR1 (N,No,R)	92	-	LC	1*	6	6	0.00353	0.16	0.08	0.19	0.48	1	62.5
**SFPQ (N,S,P,SG)**	76	2	Y	9.4	59	6	0.00367	0.68	1.79	0.90	1.52	1	6.2
**DDX5 (N,No,S)**	69		Y	9.2	57	6	0.00540	0.89	1.10	1.11	1.88	0.99	6.2
**RBMX (N,T,I)**	42	1	LC	5.6	34	6	0.00309	1.22	1.01	1.23	1.57	1	6.2
RPL26L1 (R)	17		LC	1.8	11	6	0.00664	0.64	1.73	1.69	2.73	0.99	6.1
ZC3HAV1 (N,SG)	101	-	-	1*	6	6	0.01979	0.08	0.83	0.20	0.33	1	60.0
**HNRNPU (N,P)**	91	other†	Y	10	60	6	0.00147	0.75	1.74	1.01	1.62	1	6.0
HNRNPCL1 (N)	32	1	LC	3.2	19	6	0.00044	0.66	1.53	0.82	1.53	1	5.9
RPL3 (N,No,R)	46	-	LC	3	18	6	0.00004	0.42	2.20	0.61	1.52	1	5.9
RPL14 (N,No,R)	24	-	LC	1.4	8	6	0.00037	0.61	2.42	1.09	2.06	0.99	5.9
RRBP1 (R)	152	-	LC	1*	6	6	0.01043	0.26	2.87	0.31	2.26	1	57.5
HSPA1B	70	-	-	1.4	8	6	0.0201	0.52	6.58	0.31	0.77	0.95	5.5
ZFR (N,No)	117	-	LC	1*	5.5	5.5	0.00212	0.09	0.45	0.11	0.36	1	55.0
RPS4X (N,No,R)	30	other†	-	4.8	26	5.5	0.01078	0.76	0.70	1.55	2.44	0.99	5.5
HCG26523 Unc	17	-	LC	1.6	9	5.5	0.03222	0.74	1.49	1.85	3.54	0.97	5.5

SRSF1 (N,S,P)	28	2	LC	2.2	12	5	0.01679	1.49	2.30	2.55	4.51	0.97	5.3
RPL10A (R)	25	-	-	2.6	14	5	0.00387	0.51	1.42	1.00	2.18	0.99	5.3
RPL8 (N,No,R)	28	-	-	3.4	18	5	0.015837	0.68	1.32	1.62	1.97	0.95	5.2
**RBM14 (N,No,T)**	69	2	Y	5.2	27	5	0.00030	0.87	1.00	0.86	1.74	1	5.1
RPL10 (N,R)	25	-	-	2.2	11	5	0.00510	0.36	2.14	1.20	2.18	0.99	5.0
SF3B1 (N,S)	146	-	LC	2	10	5	0.03394	0.15	0.45	0.30	0.20	0.96	5.0
B3KMC9 Unc	109	-	LC	1*	5	5	0.00502	0.60	0.70	0.72	0.80	1	50.0
SRPK1 (N,S)	74	-	LC	1*	5	5	0.04555	0.20	0.50	0.24	0.20	1	50.0
**DHX9 (N,No,T)**	141	other†	Y	9.2	43	4.5	0.00022	0.55	1.95	0.65	1.27	1	4.6
EPRS (I)	171	-	-	3	14	5	0.01973	0.22	0.51	0.31	0.80	0.94	4.6
RPL13 (N,R)	24	-	LC	4.4	20	4.5	0.0081	0.71	1.34	1.70	2.58	0.95	4.5
RPS6 (N,No,R)	29	-	LC	3.2	14	4.5	0.0075	0.91	1.09	1.26	1.96	0.97	4.5
HNRPF (N,S,P)	46	2	Y	3	13	4.5	0.00155	0.62	2.69	0.92	2.38	0.99	4.3
RPL27 (N,No,R)	16	-	LC	3.4	14	4	0.01033	0.69	0.84	1.53	2.55	0.94	4.0
**HNRNPH1 (N,No,S,P)**	51	3	Y	7	28	4	0.02574	0.64	2.00	1.10	2.30	0.93	3.7
RPS3A (N,No,R)	27	other†	-	6.6	27	4	0.02574	0.89	1.08	1.15	1.55	0.99	6.3
**HNRNPH2 (N,No,S,P,SG)**	49	3	Y	5	20	4	0.00591	0.56	1.62	1.09	2.38	0.94	4.0
RPS11 (N,No,R)	18	-	LC	4.2	16	4	0.01174	0.29	1.02	1.18	1.84	0.95	3.8
FBL (N,No,R,SG)	34	-	Y	3.6	14	5	0.00560	0.30	2.22	1.11	2.89	0.93	3.8
**TARDBP (N,S,P,SG)**	45	2	Y	1.2	4	3.5	0.00324	0.35	1.53	0.71	1.18	0.96	3.5

†other: utilizes another RNA binding motif.
PrLD predicted by PLAAC: Prion-like amino acid composition plaac.wi.mit.edu (46); LC- low complexity sequences are present such as Arg/Ser (RS domain), Arg-rich, Gly-rich Glu-rich, or elements >50aa with a low folding index.
*Peptide counts avg of 0 to 1 rounded to 1.
Bold – match data on MATR3 interactors available in public databases; https://thebiogrid.org/115126, https://www.ebi.ac.uk/intact/, http://string-db.org.
Green highlight – match with (19)
Location and function annotations derived from {uniport.org}(44, 45), (N = nuclear; No = nucleolar; S = mRNA splicing; P = RNA processing; R = ribosomal; Ch = chromatin; I = inflammation; SG = stress granule).

Supplemental Table S6. Proteins that show significantly different spectral counts for F115C-MATR3:AviTag capture.

Protein (isoform)	MW kDa	RRM	PrLD	Avg # spectra control	Avg # spectra WT	Fold Change	p Value vs Control	Avg # spectra F115C	Fold Change vs WT	p Value vs WT	G-Test vs WT	SAINT F115C	SAINT Fold
MYBBP1A (N,N,T)	149	-	-	1*	16.25	16.25	0.0006	31.67	1.95	0.04978	0.02780	1	158.3
MKI67 (N,N,C)	359	-	LC	2.20	15.50	7.05	0.09146	39.33	2.54	0.04945	0.00132	1	17.9
DDX21 (N,N,T)	87	other†	LC	3.60	25.00	6.94	0.00008	45.33	1.81	0.01357	0.01608	1	10.1
RPL5 (N,N,R)	34	-	LC	1.00	6.25	6.25	0.10384	16.67	2.67	0.03526	0.03406	1	16.7
RPL32 (R)	16	-	LC	1*	3.50	3.50	0.00999	13.67	3.90	0.02761	0.01717	1	34.2
CKAP4	66	-	Y	3.20	11.00	3.44	0.08359	25.00	2.27	0.03732	0.02155	1	7.8
Q53HW2 (R)	34	-	-	1*	3.00	3.00	0.29235	18.00	6.00	0.04108	0.00111	1	180.0
HMGB2 (N,T,C)	24	-	LC	1*	2.50	2.50	0.22817	12.67	5.07	0.02557	0.01119	1	21.1
**ALYREF (N,S,P)**	28	1	LC	1*	2.00	2.00	0.22745	10.67	5.33	0.04306	0.01933	1	13.3

†other: utilizes another RNA binding motif.

PrLD predicted by PLAAC: Prion-like amino acid composition plaac.wi.mit.edu (46); LC- low complexity sequences are present such as Arg/Ser (RS domain), Arg-rich, Gly-rich Glu-rich, or elements >50aa with a low folding index.

*Peptide counts avg of 0 to 1 rounded to 1.

Bold – match data on MATR3 interactors available in public databases; https://thebiogrid.org/115126, https://www.ebi.ac.uk/intact/, http://string-db.org.

Green – match with (19)

Location and function annotations derived from {uniport.org}(44, 45), (N = nuclear; No = nucleolar; S = mRNA splicing; P = RNA processing; R = ribosomal; Ch = chromatin; I = inflammation; SG = stress granule).

Supplemental Table S7. Proteins binding to MATR3ΔRRM2 with >3-fold enrichment relative to WT-MATR3 that are low abundance in control or WT-MATR3 samples.

Protein (location)	MW kDa	RRM	PrLD	Avg # Spectra Control	Avg # spectra WT	Avg # spectra ΔRRM2	Fold Change vs WT*	p Value vs WT	G-test Vs Control	SAINT ΔRRM2	SAINT Fold
HSPA5 (Cy)	72	-	LC	1*	1*	35.50	35.50	0.0000001	0.0000001	1	177.5
EZR (Cy)	69	-	LC	1.00	1*	28.00	28.00	0.0004464	0.0000038	1	28.0
PNPT1(Cy)	86	other†	n	1*	1*	25.50	25.50	0.0000001	0.0000006	1	127.5
PA2G4 (Cy)	44	other†	n	1*	1*	23.50	23.50	0.0004719	0.0000002	1	235.0
B7Z4V2 HSPA9 sim (N)	72	-	LC	1*	1*	20.00	20.00	0.0003250	0.0000023	1	33.3
Q59GW1 (N)	20	-	LC	1*	1.50	27.50	18.33	0.0000058	0.0000001	1	137.5
RDX (Cy)	69	-	LC	1*	1*	17.50	17.50	0.0045524	0.0004229	1	43.8
MYL6 (Cy)	17	-	n	1*	1.75	30.00	17.14	0.0010307	0.0000002	1	37.5
MSN (Cy)	68	-	LC	1*	1*	17.00	17.00	0.0018334	0.0005652	1	28.3
CYFIP1 (Cy)	145	-	n	1*	1.25	19.00	15.20	0.0001182	0.0001758	1	47.5
RPLP1 (Cy)	12	-	n	1*	1*	15.00	15.00	0.0000424	0.0004304	1	75.0
NCKAP1 (Cy)	130	-	n	1*	1*	14.50	14.50	0.0000001	0.0000903	1	145.0
NOLC1 (N,Cy)	75	-	LC	1*	1*	14.50	14.50	0.0000186	0.0000903	1	36.3
CYFIP2 (N,Cy)	143	-	n	1*	1.25	17.00	13.60	0.0000066	0.0005652	1	42.5
D3DQY9 (N)	22	-	LC	1*	1*	13.50	13.50	0.0000001	0.0001760	1	135.0
HEL-S-2a	22	-	n	1*	1*	13.50	13.50	0.0000014	0.0001760	1	22.5
PSPC1 (N,Cy)	59	2	Y	1*	1.25	16.50	13.20	0.0009990	0.0066882	1	20.6
A8K245 (N,Cy)	45	-	LC	1.00	1*	12.50	12.50	0.0038108	0.0440165	1	12.5
EIF5A (N,Cy)	16	-	n	1*	1*	12.50	12.50	0.0000393	0.0019814	1	31.3
FEN1 (N)	43	-	LC	1.00	1.00	12.50	12.50	0.0015825	0.0440165	1	12.5
NME1 (N,Cy)	33	-	n	1*	1*	12.50	12.50	0.0010837	0.0003423	1	125.0
CBX3 (N)	21	-	LC	1*	1.00	12.00	12.00	0.0000933	0.0004771	1	120.0
SSBP1 (Cy)	17	-	n	1*	1*	12.00	12.00	0.0005500	0.0251027	1	20.0
B4DNN4 (N)	86	-	LC	1*	1*	11.50	11.50	0.0000001	0.0006647	1	115.0
CFL1 (N,Cy)	19	-	n	1*	1*	11.50	11.50	0.0000459	0.0006647	1	57.5
ALDOA (Cy)	40	-	n	1*	1*	11.00	11.00	0.0000594	0.0161880	1	13.8
EIF2S2 (Cy)	38	-	LC	1*	1.50	16.50	11.00	0.0002966	0.0000237	1	165.0
HCFC1 (N,Cy)	213	-	n	1*	1*	11.00	11.00	0.0034757	0.0009255	1	110.0
H0YHG0 (N)	59	-	LC	1*	1*	10.50	10.50	0.0000023	0.0012881	1	52.5
MTHFD1L (Cy)	99	-	LC	1*	1.25	13.00	10.40	0.0007487	0.0002455	1	32.5
**ALYREF (N,Cy)**	28	1	LC	1*	2.00	20.50	10.25	0.0000066	0.0002808	1	25.6
DNM3 (Cy)	97	-	n	1*	1*	10.00	10.00	0.0025635	0.0274882	1	25.0
FKBP3 (N,Cy)	25	-	LC	1*	1*	9.50	9.50	0.0016671	0.0024909	1	23.8
KIF4A (N,Cy)	128	-	LC	1*	1*	9.50	9.50	0.0000718	0.0024909	1	95.0
I6TRR8 (N)	109	-	n	1*	1*	9.00	9.00	0.0028852	0.0158264	1	45.0
IFIT1 (Cy)	52	LC	n	1*	1.00	9.00	9.00	0.0000129	0.0034605	1	90.0
LBR (N)	71	-	LC	1*	1.75	15.50	8.86	0.0013488	0.0108830	1	19.4
VDAC1 (Cy)	31	-	n	1.60	1.75	15.50	8.86	0.0003094	0.0108830	1	9.7
FLNC (Cy)	287	-	n	1*	1*	8.50	8.50	0.0013559	0.0048041	1	85.0
MRE11A (N)	81	-	LC	1*	1*	8.50	8.50	0.0001230	0.0048041	1	85.0
DKFZp586K0821 unc	22	-	-	1*	1*	8.00	8.00	0.0009005	0.0066643	1	40.0
DYNC1LI1 (Cy)	57	-	-	1*	1*	7.50	7.50	0.0003758	0.0374259	1	37.5
IMPDH2 (N,Cy)	51	-	-	1*	1*	7.50	7.50	0.0000010	0.0092372	1	75.0
MAP1B (Cy)	271	-	LC	1*	1*	7.50	7.50	0.0000125	0.0374259	1	37.5
A0A024RB41 (Cy)	23	-	LC	1*	1*	6.50	6.50	0.0000021	0.0176967	1	65.0
SMARCC2 (N)	133	-	Y	1*	1*	6.50	6.50	0.0004417	0.0176967	1	65.0
Q69YJ6 unc	84	-	LC	1*	1*	6.00	6.00	0.0000933	0.0244555	1	60.0
RCN2 (Cy)	39	-	LC	1*	1.25	7.50	6.00	0.0000010	0.0092372	1	75.0
DDOST (Cy)	51	-	n	1*	1.75	10.00	5.71	0.0001073	0.0274882	1	16.7
SND1 (N,Cy)	102	-	n	1*	2.25	12.50	5.56	0.0010837	0.0003423	1	125.0
ENAH (Cy)	66	-	LC	1*	1*	5.50	5.50	0.0000048	0.0337546	1	60.0
PECI (Cy)	40	-	n	1*	1*	5.50	5.50	0.0000048	0.0337546	1	55.0
DDX6 (N,Cy)	54	LC	Y	1*	2.75	14.00	5.09	0.0000767	0.0092042	1	23.3
ACSL3 (Cy)	80	-	n	1*	2.50	12.50	5.00	0.0016593	0.0071805	1	31.3

†other: utilizes another RNA binding motif.

PrLD predicted by PLAAC: Prion-like amino acid composition plaac.wi.mit.edu (46); LC- low complexity sequences are present such as Arg/Ser (RS domain), Arg-rich, Gly-rich Glu-rich, or elements >50aa with a low folding index.

*Peptide counts avg of 0 to 1 rounded to 1.

Bold – match data on MATR3 interactors available in public databases; https://thebiogrid.org/115126, https://www.ebi.ac.uk/intact/, http://string-db.org.

Green – match with (19)

Location annotations derived from {uniport.org} (N = nuclear; Cy = cytoplasmic).
